# Agarose-free protocol for preparing human organotypic lung cultures to study respiratory virus infection and evaluate antivirals *ex vivo*

**DOI:** 10.1016/j.xpro.2026.104896

**Published:** 2026-10-13

**Authors:** Lola Canus, Florentine Jacolin, Virginie Vasseur, Adeline Cezard, Eva Ogire, Anne Aublin-Gex, Amelie Bourgeais, Camille David, Alexandra Erny, Fabienne Archer, Antoine Legras, Damien Sizaret, Antoine Guillon, Vincent Lotteau, Pierre-Olivier Vidalain, Mustapha Si-Tahar, Laure Perrin-Cocon, Cyrille Mathieu

**Affiliations:** 1 CIRI, Centre International de Recherche en Infectiologie, Team NeuroInvasion, Tropism and Viral Encephalitis, University Lyon, Inserm, U1111, CNRS, UMR5308, Université Lyon 1, Ecole Normale Supérieure de Lyon, 69007 Lyon, France; 2 CIRI, Centre International de Recherche en Infectiologie, Team Viral Infection, Metabolism and Immunity, University Lyon, Inserm, U1111, CNRS, UMR5308, Université Lyon 1, Ecole Normale Supérieure de Lyon, 69007 Lyon, France; 3 Inserm, U1100, Centre D’Etude des Pathologies Respiratoires (CEPR), University of Tours, 37000 Tours, France; 4 IVPC, Infections Virales et Pathologie Comparée, UMR754, INRAE, Université Lyon 1, EPHE, 69007 Lyon, France; 5 Service de Chirurgie Thoracique, CHRU de Tours, Université de Tours, Tours, France; 6 Service de Pathologie, CHRU de Tours, Tours, France; 7 Service de Médecine Intensive -Réanimation, CHRU de Tours, Tours, France; 8 Laboratoire P4 INSERM-Jean Mérieux, Lyon, France

**Keywords:** Cell culture, Cell isolation, Health Sciences, Immunology, Microbiology, Tissue Engineering

## Abstract

Here, we present a protocol to generate human organotypic lung cultures from surgical lung resection for the study of respiratory infections. We describe steps for tissue harvesting, biopsy punching, mechanical slicing, and culture at the air-liquid interface. This technique enables robust *ex vivo* infections of human lung tissues with respiratory viruses, including influenza A and Nipah. It can be used to study host-pathogen interactions, analyze innate immune responses, and evaluate antiviral candidates in physiologically relevant human lung tissue.

For complete details on the use and execution of this protocol, please refer to Cezard et al.^1^

## Highlights

• Steps for human organotypic lung cultures from surgical lung resection
• Guidance on punch biopsies and mechanical slicing of the lung tissue
• Instructions for culture at the air-liquid interface
• Procedures ex vivo infections with respiratory viruses

## Abstract (editor highlights)

Publisher’s note: Undertaking any experimental protocol requires adherence to local institutional guidelines for laboratory safety and ethics.

## Graphical abstract

**Figure:**
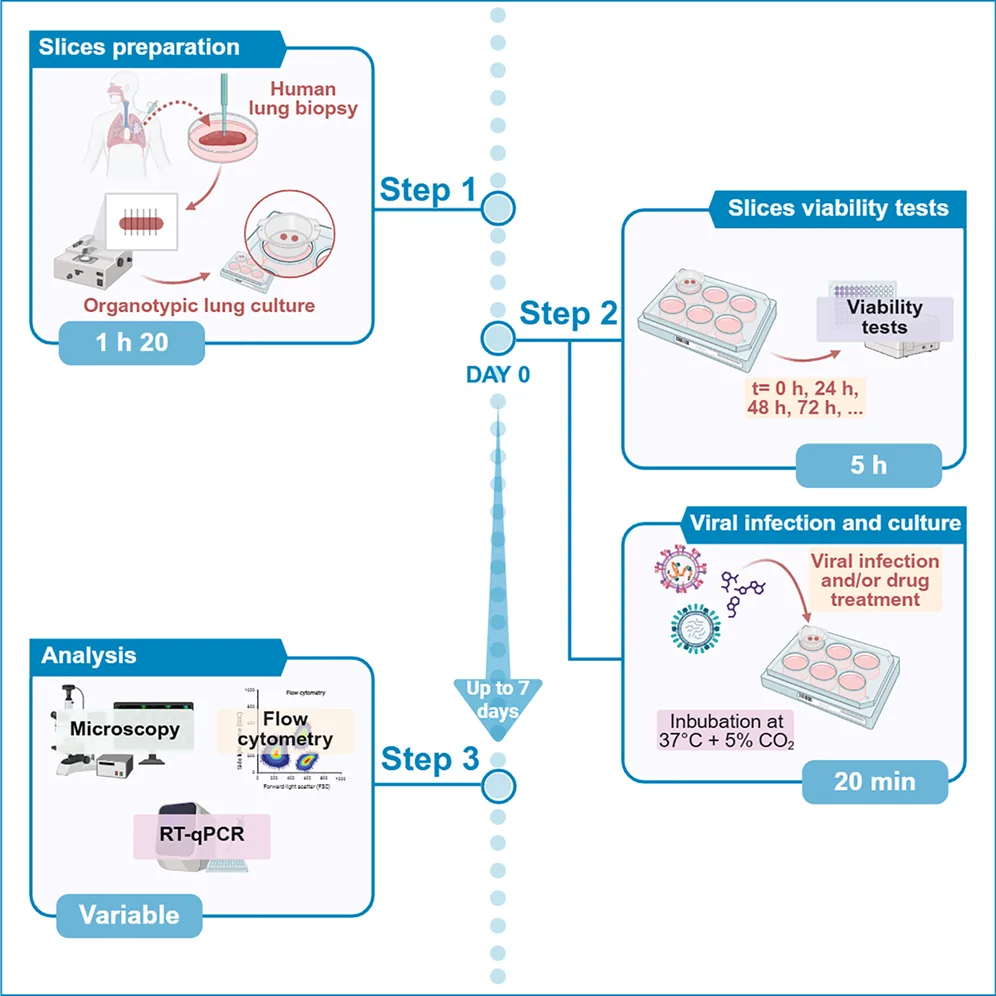

## Before you begin

According to the World Health Organization, airborne viral infections are among the leading causes of human disease worldwide, with lower respiratory tract infections ranking among the deadliest. Understanding respiratory virus infection and host response at the tissue level remains challenging due to the limitations of experimental models. *In vivo* animal models, such as mice and hamsters, are widely used but often remain poorly suited for tissue-specific mechanistic investigations and are constrained by experimental complexity and ethical considerations. In contrast, *in vitro* systems enable controlled experimentation but fail to recapitulate the three-dimensional architecture and cellular diversity of the lung. Organotypic lung cultures therefore represent a powerful *ex vivo* alternative, as they preserve native tissue organization and, when maintained at the air–liquid interface, reproduce key physiological features of the respiratory epithelium. We have previously established standardized organotypic cultures from animals, including brain substructures, lung, kidney, and liver for infection studies. Our models enable reproducible *ex vivo* investigations of zoonotic infections and provide insights into tissue-specific viral tropism and innate immune responses while contributing to the reduction of animal experimentation.^2^^,^^3^^,^^4^^,^^5^^,^^6^^,^^7^^,^^8^^,^^9^^,^^10^ Building on this experience, we have implemented this approach using human lung tissue.

This protocol describes a rapid and standardized method to generate and maintain human organotypic lung cultures (huOLC) from surgical resections and how to infect huOLC with various respiratory pathogens such as Influenza virus and Nipah virus. Compared with previously reported protocols to prepare precision-cut lung slices (PCLS), our method introduces and combines several key innovations designed to improve tissue survival, metabolic respiration, and the maintenance of resident immune cells for at least the duration of infection studies.^11^ It details key steps from tissue coring and slicing, huOLC maintenance, and infection with BSL-2 and BSL-4 pathogens with the output tests detailed to follow in-depth examination of viral infection dynamics, host tissue response as well as the evaluation of antivirals. Please note that this protocol necessitates proper institutional and ethical approval prior to working with human tissue samples.

### Innovation

This workflow describes an agarose-free protocol to generate huOLCs from fresh surgical resections. Unlike many studies in which the lung tissue is inflated and embedded in low-melting agarose (1–3%) prior to slicing with vibratome or Krumdieck slicers,^12^^,^^13^^,^^14^^,^^15^^,^^16^^,^^17^^,^^18^^,^^19^^,^^20^^,^^21^^,^^22^ our protocol enables immediate processing and thus significantly accelerates the experimental workflow. Tissue slicing is performed using a tissue chopper, preserving native tissue organization, while culture of huOLCs at the air-liquid interface on microporous and hydrophobic membranes^23^ better mimics physiological lung conditions *in vivo*. This configuration allows basolateral diffusion of nutrients while maintaining exposure of the apical surface to air. Unlike submerged culture conditions commonly used for PCLS,^12^^,^^15^^,^^16^^,^^17^^,^^18^^,^^20^^,^^21^^,^^22^^,^^24^ this organotypic lung slices approach limits oxygen deprivation and reduces the loss of non-adherent cells, supporting sustained structural integrity and viability for at least 6 days of culture (Figure 1).

**Figure 1:**
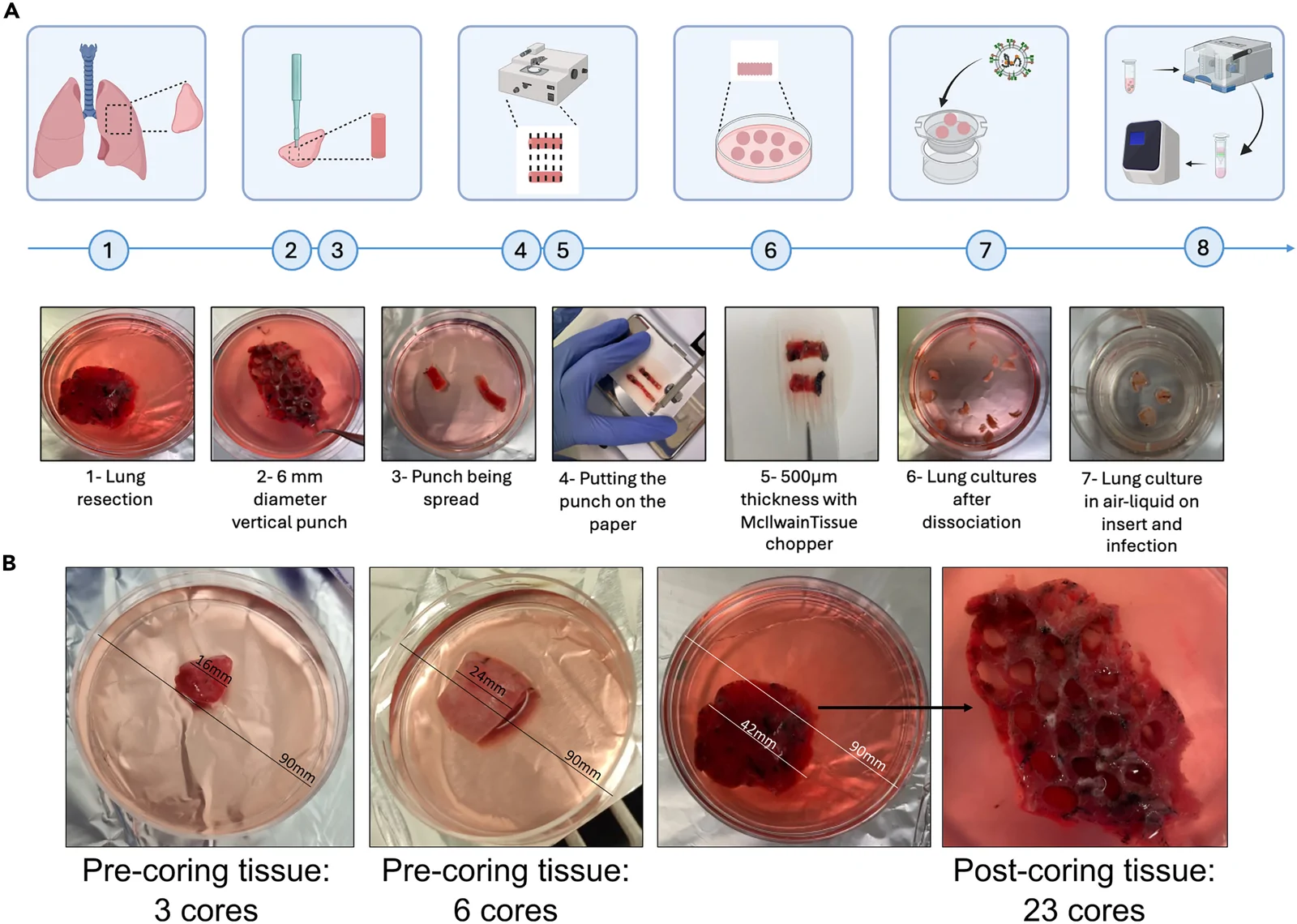
Slicing procedure (A) Schematic slicing procedure. Created in BioRender. MATHIEU, C. (2025) https://BioRender.com/v34z384. (B) Representative human lung tissue samples used for huOLCs generation and corresponding coring output. Three human lung tissue specimens are shown in 90 mm Petri dishes, with the approximate diameter of each sample (in mm) indicated. The number of cylindrical cores obtained from each tissue is reported for each specimen. The right panel additionally shows the third tissue sample after coring, illustrating the remaining material following core extraction.

Agarose-based methods require forced lung inflation, which can alter the native architecture, induce mechanical stress, cause microstructural damage, and trigger inflammatory responses. This deviates from the physiological state that PCLS or huOLC models are intended to preserve.

Moreover, the pulmonary immune system includes non-adherent cells, including alveolar macrophages. Agarose embedding, filling the alveolar spaces, which is subsequently liquefied and released into the culture medium is causing a washing effect that can result in the loss of these immune cells. Consequently, agarose-based protocols may alter both cellular composition and the immune landscape of the tissue, representing a significant limitation for studies investigating host-pathogen interactions. Agarose is used only for mechanical support, enabling sectioning with vibratome or microtome. Our workflow, using a tissue chopper, avoiding agarose embedding, preserves native structural and cellular integrity, and allows immediate processing of freshly resected samples.

Beyond biological considerations, the agarose-free approach offers practical advantages: agarose inflation is technically demanding and introduces variability (operator handling, temperature, embedding efficiency). Eliminating this step makes our protocol simpler, faster, and more reproducible, enabling rapid experiment initiation shortly after collection, limiting early tissue adaptation or repair responses while preserving conditions more closely reflecting the native in vivo state. It is also compatible with human lung tissue obtained from surgical resections, where whole lungs are unavailable, making tracheal inflation impossible and the agarose technique impractical.

Overall, our agarose-free workflow offers a complementary approach prioritizing native tissue structure, cellular composition, and physiological relevance, while improving experimental efficiency.

In previous studies, PCLS have been maintained in various culture media. Dulbecco’s Modified Eagle Medium/F12 (DMEM/F12)^13^^,^^14^^,^^15^^,^^16^^,^^23^ or HAM (DMEM/F12 HAM)^17^^,^^18^^,^^19^^,^^20^^,^^21^ formulations generally support tissue viability and structural integrity, whereas Minimum Essential Medium (MEM) has been reported as less suitable for long-term maintenance of complex lung architecture.^24^ In some studies, fetal bovine serum (FBS) is added at low concentrations (typically 0.1%) to help preserve cell composition from cell differentiation. Here, we have chosen to culture huOLCs at the air-liquid interface to avoid hypoxia related to slices immersion and to use a serum-free 1:1 mixture of Advanced DMEM/F-12 and Bronchial Epithelial Growth Medium (BEGM), the latter being specifically formulated for airway epithelial cells. This combination provides optimal epithelial preservation while maintaining low basal secretion of inflammatory interleukin (IL)-6 making it particularly suited for infection studies.^25^

### Institutional permissions

Human lung tissue samples were obtained from patients undergoing lung resection at two French hospitals; the Centre Hospitalier Régional Universitaire de Tours (CHRU), and the Hospices Civils de Lyon (HCL) in accordance with applicable ethical requirements. Informed consent was obtained from all patients in accordance with the Helsinki Declaration and the ethical principles set out in the Belmont Report of the U.S. Department of Health and Human Services.

Tissue and cell collections were declared to the French Ministry of Higher Education, Research, and Innovation (MESRI, authorization DC-2008–308).

For sample obtained at the CHRU, all experimental procedures complied with the Code of Ethics of the World Medical Association and were approved by the Ethics Committee of the CHRU of Tours.

For the samples obtained at the HCL, human biological samples and associated data were obtained from the HCL Biobank (CRB-HCL BB-0033–00046).

The implementation of this protocol requires authorization from the local authorities and compliance with their guidelines.

### Patients’ information and criteria of inclusion and exclusion

We have collected lung tissues from patients who underwent surgery with lung resection. Lung tissue samples were collected at a distance from the primary lesion (most frequently malignant). Tumor-free resection margins were confirmed by intraoperative section analysis. Inclusion criteria were: adult patients scheduled for lobectomy, segmentectomy, or wedge resection, and provision of informed consent. Exclusion criteria included suspected or confirmed active pulmonary infection within the 48 hours preceding surgery, defined by the presence of one or more of the following: (1) clinical symptoms (cough, sputum production, fever, dyspnea, or chest pain); (2) elevated inflammatory biomarkers (C-reactive protein [CRP], procalcitonin [PCT], or neutrophilia); and/or (3) a newly abnormal chest X-ray demonstrating lung consolidation. The main clinical and pathological characteristics of the two cohorts (Tours and Lyon) are summarized in Table 1.

**Table 1:** Patients’ characteristics

Cohorts	TOURS	LYON
Patients’ characteristics	(n=8)	(n=6)
Sex, n male (%)	5 (62.5)	1 (16.6)
Age (year)	67.7 ± 12.4	56.2 ± 9.8
Body Weight (kg)	81.3 ± 14.4	-
Body Mass Index (kg/m2)	27 ± 3.5	-
Active smokers, n (%)	1 (13)	0
Former smokers, n (%)	5 (63)	3 (50)
Chronic obstructive pulmonary disease, n (%)	1 (13)	1 (16.6)
Asthmaticus	1 (13)	0
Respiratory function parameters		
Forced expiratory volume in the first second (% of predicted)	87.7 ± 10.2	-
Tiffeneau index (%)	83.4 ± 17.7	-
Diffusing capacity of the lung for carbon monoxide (DLCO, %)	72 ± 12	-

Quantitative data are reported as the mean ± SD; qualitative value are reported as n (%).

### Preparation of the culture plates with inserts

**Timing: About 10 min per 6-well plate under biosafety cabinet in the BSL-2 for steps 1–3**

1. Remove the lid of a sterile 6-well plate and add 1 mL of huOLC culture medium (1:1 mixture of supplemented BEGM and Advanced medium) to each well.
2. Open the packaging of the polytetrafluoroethylene (PTFE) membrane inserts under sterile conditions.

**CRITICAL:** Step 1 and 2 should be performed one day before tissue slicing.

3. Using straight and sterile tweezers, gently place one insert into each well, ensuring it lands on the surface of the medium.

***Note:*** This step helps reverse the hydrophobicity of the PTFE membrane, allowing proper contact with the medium.

**CRITICAL:** For this step, ensure no air bubbles are trapped between the PTFE membrane and the medium, as this can impair reversion of the hydrophobicity of the membrane and liquid absorption by cultures throughout micropores, and *in fine* affecting tissue viability and nutrients diffusion.

**Pause point:** Inserts can be prepared in advance (e.g., on Friday for an experiment on Monday). If prepared more than 24 h in advance, replace the medium to maintain optimal conditions.

***Note:*** PTFE membranes were used instead of PET membranes because their chemical and optical properties allow direct microscopic observation (notably fluorescence microscopy) and do not affect the observation of the IP stanning.^2^

4. Incubate the prepared 6-well plate for 12 hours at 37 °C in a humidified incubator with 5% CO_2_.

### Slicing preparation

**Timing: About 30 min for steps 5–13**

5. Decontaminate the laboratory bench thoroughly using 70% ethanol (EtOH).
6. Cover the bench with aluminum foil and spray it with 70% EtOH. Allow the aluminum foil to dry.

**CRITICAL:** Place the aluminum foil with the dull side facing down to ensure proper surface contact.

***Note:*** The bench can be prepared the day before. Use of aluminum foil is only required when preparation is not performed in a clean white room.

7. Sterilize all slicing and dissection equipment with 70% EtOH, i.e., tissue chopper, Leica microscope. All the equipment must be fully dry before use.
8. Prepare a container filled with 70% EtOH for decontamination of dissection material before use (i.e., razor blades, scissors, curved tweezers, straight tweezers). After decontamination, put dissection tools on a sterile petri dish to dry before use.

***Note:*** Dissection tools may be autoclaved if additional sterilization is required. In our experience, cleaning with 70% EtOH is sufficient to maintain sterile conditions; however, sterilization procedures may vary between laboratory depending on contamination risk.

9. Prepare the Whatman paper squares (6 cm x 6 cm).

**CRITICAL:** Whatman paper must be autoclaved before use.

***Note:*** Autoclaving can be performed in advance and stored in a sterile container until use.

10. Prepare the slicing medium by combining Hibernate A + 10,000 U/mL Penicillin/Streptomycin in a 50 mL sterile falcon tube (99% Hibernate-A supplemented with 1% penicillin-streptomycin).
11. Aliquot 5 mL of slicing medium in 35 mm sterile petri dishes and keep the lid on the dishes as long as possible to avoid contamination.

**CRITICAL:** Store the remaining slicing medium at 4°C.

12. Prepare the transfer pipette by cutting the conical extremity of a 5 mL sterile plastic pipette, remove the filter without touching the pipette and insert the top part into the pipette bulb (Figure 2).

**Figure 2:**
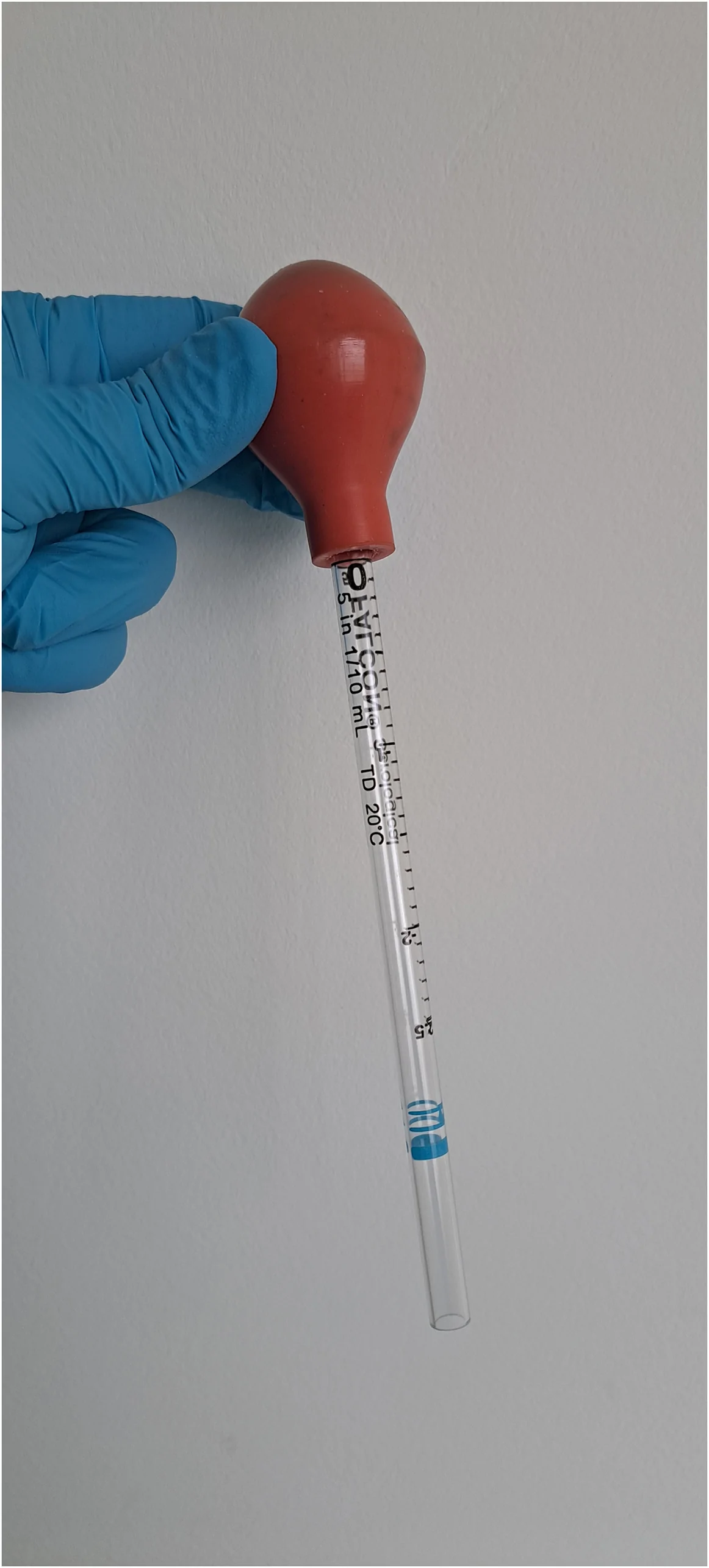
Picture of the transfer pipette system
13. Set up the tissue chopper by adjusting the slicing parameters for the desired thickness (here 500 μm), blade force and speed.

**CRITICAL:** Razor and scalpel blades are extremely sharp. Handle with care and wear appropriate protective equipment.

## Key resources table

**Table:** 

REAGENT or RESOURCE	SOURCE	IDENTIFIER
**Antibodies**		

Rabbit anti-Influenza nucleoprotein primary antibody, used as 1:400 dilution	Thermo Fischer Scientific	Cat#PA5-32242
Goat anti-rabbit Alexa A488 conjugated secondary antibody, Used as 1:1000 dilution	Thermo Fischer Scientific	Cat#A-11034
Anti-Influenza A Virus Nucleoprotein antibody (FITC) Used as 1:50	Abcam	Cat#ab20921
Hoechst solution, used as 1:000 dilution	Thermo Fischer Scientific	Cat#62249
Propidium Iodide	Sigma-Aldrich	Cat#P4170-25MG
EpCAM, Used as 1:200 dilution	Biolegend	Cat#324206
CD31, Used as 1:200 dilution	Biolegend	Cat#561653
CD45, Used as 1:400 dilution	Biolegend	Cat#368524
LIVE/DEAD™ Fixable Red Dead Cell Stain Kit, Used as 1:1000 dilution	Thermo Fischer Scientific	Cat#L23102
Anti-tubulin Antibody Used as 1:500 dilution	Sigma Aldrich	Cat#T6793
Anti-mouse-AF647 Used as 1:500 dilution	Thermo Fischer Scientific	Cat#A21242

**Bacterial and virus strains**		

Mouse-adapted influenza A/Scotland/20/74 (H3N2) virus	Pr. Sylvie van der Werf’s team; Pasteur Institute, Paris, France	N/A
Recombinant Nipah Malaysia virus (rNiV-EGFP) isolate UMMC1	INSERM Jean Mérieux BSL4, Lyon^26^	GeneBank AY029767 Yoneda et al*.*,^26^

**Biological samples**		

Human lung resection	Centre Hospitalier Régional Universitaire de Tours	N/A
Human lung resection	Hospices Civils de Lyon	N/A

**Chemicals, peptides, and recombinant proteins**		

Advanced DMEM/F-12	Thermo Fischer Scientific	Cat#12634010
Hibernate™-A medium	Thermo Fischer Scientific	Cat# A1247501
MEM medium, with glucose and Glutamax	Gibco	Cat#41090036
Fetal Bovine serum (FBS)	Biosera	Cat# FB1285
Dulbecco’s phosphate buffer saline 1X (DPBS 1X), w/o calcium/magnesium	Thermo Fischer Scientific	Cat#14190094
BEGM® Bronchial Epithelial Cell Growth Medium BulletKit	Lonza	Cat#CC-3170
HEPES	Thermo Fischer Scientific	Cat#15630056
Red Blood Cell Lysis Buffer	Gibco	Cat#A1049201
Penicillin/Streptomycin	Thermo Fischer Scientific	Cat#15140122
Primocin	InvivoGen	Cat#ant-pm-05
Formaldehyde 20% sol. Em grade	LFG distribution	Cat# EM-15713
Crystal Violet	Sigma	Cat# C3886
Ethylenediaminetetraacetic acid (EDTA)	Sigma Aldrich	Cat# E9884
Methanol	VWR	Cat#20856.296
Ethanol absolute	VWR	Cat#20821.296P
Trizol	Thermo Fischer Scientific	Cat#15596026
Tri Reagent	Euromedex	Cat#TR118-100
Thiazolyl Blue Tetrazolium Bromide (MTT)	Sigma Aldrich	Cat# M2128
Tris-HCl	Sigma Aldrich	Cat# T5941
Glycerol	Sigma Aldrich	Cat# G5516
Bovine serum albumin (BSA)	Sigma Aldrich	Cat# A8806
Triton X-100	Sigma Aldrich	Cat# X100-100ML
Liberase,	Roche	Cat#05401020001
Dispase II	Roche	Cat#4942078001
DNAse	Roche	Cat#11284932001
Baloxavir marboxil	MedChemExpress	Cat#HY-109025A
TPCK-treated trypsin	Sigma Aldrich	Cat#T1426-MG

**Critical commercial assays**		

Celsior solution	Institut Georges Lopez	Cat#533682
iScript cDNA Synthesis kit	Bio-Rad	Cat#1708891BUN
High-Capacity RNA-to-cDNA kit	Thermo Fisher Scientific	Cat# 4368814
Platinum SYBR Green qPCR SuperMix-UDG	Invitrogen	Cat# 11733046
Direct-zol RNA Microprep Kits	Zymo	Cat# R2062
LDH-Glo Cytotoxicity assay	Promega	Cat# J2380
Cytometric Bead Array (CBA) Master Buffet Kit	BD Biosciences	Cat#558265
CBA Assay Diluent	BD Biosciences	Cat#560104
BD™ CBA Human IL-8 Flex Set	BD Biosciences	Cat#558277
BD™ CBA Human IP-10 Flex Set	BD Biosciences	Cat#558280
BD™ CBA Human IL-6 Flex Set	BD Biosciences	Cat#558276
BD™ CBA Human MCP-1 Flex Set	BD Biosciences	Cat#558287

**Experimental models: Cell lines**		

Madin-Darby Canine Kidney (MDCK)	ATCC®	RL-2935

**Oligonucleotides**		

NiV-N For: 5′ GGCAGGATTCTTCGCAACCATC 3′	Mathieu et al.,^27^	N/A
NiV-N Rev: 5′ GGCTCTTGGGCCAATTTCTCTG 3′	Mathieu et al.,^27^	N/A
human GAPDH For: 5′ CACCCACTCCTCCACCTTTGAC 3′	Mathieu et al.,^27^	N/A
human GAPDH Rev: 5′ GTCCACCAC CCTGTTGCTGTAG 3′	Mathieu et al.,^27^	N/A
IAV-M For: 5′ AAGACCAATCCT GTCACCTCTGA 3′	Guillon et al.,^28^	N/A
IAV-M Rev: 5′ CAAAGCGTCTACGCTGCAGTCC 3′	Guillon et al.,^28^	N/A
human Rplp0 For: 5′ CACTGA GATCAGGGACATGTTG 3′	Jacolin et al.,^10^	N/A
human Rplp0 Rev: 5′ CTTCACATGGGGAATGG 3′	Jacolin et al.,^10^	N/A

**Software and algorithms**		

Graphpad Prism 8 or later	GraphPad Software	https://www.graphpad.com
StepOne Plus Real-Time PCR System Software	Applied Biosystems (ThermoFisher Scientific)	https://www.thermofisher.com/fr/fr/home/technical-resources/software-downloads/StepOne-and-StepOnePlus-Real-Time-PCR-System.html
BioRender	BioRender	https://www.biorender.com
FlowJo V10	BD Biosciences	https://www.flowjo.com/flowjo/download
FIJI (ImageJ)	NIH	https://imagej.net/software/fiji/
Beacon Designer (version 8)	Premier Biosoft	https://www.premierbiosoft.com/molecular_beacons/

**Other**		

Mcllwain Tissue Chopper	Campden Instruments	Cat# MIK 112116
Stereomicroscope for dissection	Leica	Cat# Leica LED2000
TissueLyser II	Qiagen	Cat#85300
Precellys	Bertin technologies	Cat#10144–570
Sterile Falcon 6-well flat bottom plate	Corning, Falcon®	Cat#353046
Millicell Cell Culture Insert, 30 mm, hydrophilic PTFE, 0.4 μm	Sigma Aldrich	Cat# PICM0RG50
Whatman™ 3MM CHR Chromatography Paper	Dominique Dutscher	Cat#3030–917
TC-treated cell culture Petri dish, Sterile	Corning, Falcon®	Cat#353003
Sterile Petri dishes, 35 mm	Corning, Falcon®	Cat#351008
Biopsy punch 6 mm	Kai medical	Cat# BP-60F/48601/LCH-PUK-60
Blade Gillette, platinum	Monoprix	N/A
Stainless steel dissecting scissors length 11 cm	Dominique Dutscher	Cat#005064
Straight tweezers type n°5 length 11 cm	Dominique Dutscher	Cat#005092
Curved tweezers type n°7 length 11 cm	Dominique Dutscher	Cat#005093
Sterile scalpel single use lame N°10	PARAMOUNT	Cat#90189084
Conical 50 mL (bulk) Falcon tubes	Dominique Dutscher	Cat#352070
Pipette for cell culture 5 mL Falcon	Dominique Dutscher	Cat#357543
1,000 μL XL graduated TipOne® Filter Tips, Natural (sterile)	Starlab	Cat# S1122-1730
200 μL UltraPoint® graduated TipOne® Filter Tips, Natural, Refills (sterile)	Starlab	Cat# S1123-8710
20 μL UltraPoint® graduated TipOne® Filter Tips, Natural, Refills (sterile)	Starlab	Cat# S1123-1710
10/20 μL XL graduated TipOne® Filter Tips, Refills (sterile)	Starlab	Cat# S1120-3710
Filter tips 0,1–10 μL microguard 10g low binding cell rack sterile 96 multi x 960	Sorenson	Cat#35200
Non-sterile stainless-steel beads, ø 3.2 mm	Dominique Dutscher	Cat#152039
Microtubes Safe-Lock 1,5 mL PCR clean x 1000	Eppendorf	Cat#0030123328
Microtubes Safe-Lock 2,0 mL x 1000	Eppendorf	Cat#0030123344
2 mL tubes with O-ring for Precellys use	Bertin technologies	Cat#50–154–2961
Pipette bulb	N/A	N/A
5% CO_2_ and 37°C incubator	N/A	N/A
Biosafety cabinet (BSL-2 and BSL-4)	N/A	N/A
70% ethanol (H20 + Ethanol absolute)	VWR	Cat#20821.296P
Aluminum foil	N/A	N/A
AcroPrep Advance 96well-plates (PALL®), multi-well filter plates for solvent filtration 1.2 μm, 350 μL	Cytiva	Cat# PN5039
StepOnePlus™ Real-Time PCR System	Thermo Fisher Scientific, Applied Biosystems™	Cat#4376600)
pH-meter	Mettler Toledo	N/A
LSR Fortessa II flow cytometer	BD Biosciences	N/A
CytoFLEX	Beckman Coulter	N/A
Luminescence, fluorescence Tristar 5 microplate reader	Berthold	Cat#Tristar 5 Multimode Reader
Multi-well plate vacuum manifold PALL® 5017	Cytiva	Cat#17124461
20G needle	BD Biosciences	Cat#305175

## Materials and equipment

**Table:** Supplemented BEGM

Reagent	Final concentration	Amount
BEBM Basal Medium	N/A	500 mL
Bovine Pituitary Extract	52 μg/mL	2 mL
Insulin	5 μg/mL	500 μL
Hydrocortisone	0.5 μg/mL	500 μL
Gentamicin/Amphotericin-1000	Gentamicin: 30 μg/mL Amphotericin: 15 ng/mL	500 μL
Retinoic acid	0.1 ng/mL	500 μL
Transferrin	10 μg/mL	500 μL
Triiodothyronine	6.5 ng/mL	500 μL
Epinephrine	0.5 μg/mL	500 μL
Human Epidermal Growth Factor	0.5 ng/mL	500 μL
**Total**	**N/A**	**506 mL**

N/A = not applicable.

***Note:*** Divide the supplemented medium into 50 mL aliquots and store at −80°C for several months.

### Supplemented advanced DMEM/F12

Add 5 mL of Glutamax and 5 mL of HEPES to 500 ml of Advanced DMEM/F12. Store at 4°C for up to 3 months.

### huOLC culture medium

Mix 25 mL of supplemented BEGM with 25 mL of supplemented Advanced DMEM/F12. Store up to 3 weeks at 4°C.

**Table:** MDCK (Madin-Darby Canine Kidney) complete culture medium

Reagent	Final concentration	Amount
MEM	N/A	500 mL
Penicillin/Streptomycin	1%	5 mL
FBS	10%	5 mL
**Total**	**N/A**	**510 mL**

Store at 4°C up to 6 months.

### MDCK infection medium

Add 1% of Penicillin/streptomycin 100X to MEM medium. TPCK-treated trypsin is added extemporaneously with a final concentration of 1 μg/mL.

### Penicillin/streptomycin 100×

Once you receive the Penicillin/streptomycin aliquot it and thaw the day you need it. **Note:** Aliquot in 5 mL and store at −20°C until thawing process.

### Dissection/slicing medium

Prepare 50 mL of Hibernate®-A supplemented with 500 μL of Penicillin/streptomycin 100X (99% Hibernate-A supplemented with 1% penicillin-streptomycin). Store at 4°C and only on the day of slicing (no long-term storage at 4°C or freezing possible).

### Formaldehyde 4%

Under a fume hood, dilute to 4% with DPBS 1X (10 mL of 20% formaldehyde with 40 mL of DPBS 1X). Store at −20°C until thawing process.

**CRITICAL:** Carcinogenic, mutagenic or toxic properties.

### Solution to saturate aldehyde groups after huOLC fixation

Weigh 0.03 g glycine then add 30 mL DPBS 1X. Store at 4°C for up to 1 year.

### Permeabilization solution for immunostaining

In 25 mL DPBS 1X, add 125 μL Triton X-100 to a final concentration of 0.5%. Store at 4°C for up to 1 year and mix before use.

**Table:** Blocking solution for immunostaining

Reagent	Final concentration	Amount
DPBS 1X	1X	20 mL
BSA	1%	0.2 g
Triton X-100	0.5%	100 μL
**Total**	**N/A**	**20 mL**

Store at 4°C for up to 6 months and mix before use.

### Lactate dehydrogenase storage buffer

LDH storage buffer before is prepared according to manufacturer instructions.

**Table:** 

Reagent	Final concentration	Amount
Milli-Q water	N/A	180 mL
Tris-HCl	200 mM	6.304 g
Glycerol	10%	20 mL
BSA	1%	2 g
**Total**	**N/A**	**200 mL**

N/A = not applicable.

***Note:*** Adjust pH after addition of Tris-HCl to pH 7.3. Store at 4°C, for up to 6 months.

**Table:** Cytoflex digestion buffer

Reagent	Final concentration	Amount
DPBS 1X	N/A	470 μL
Dispase II	5 mg/mL	2.5 mg
Liberase	0.125 mg/mL	25 μL
DNase	0.1 mg/mL	5 μL
**Total**	**N/A**	**500 μL**

Store at 4°C for up to 24 h.

**Table:** Crystal Violet

Reagent	Final concentration	Amount
Crystal Violet	0.3%	3 g
Milli-Q water	N/A	800 mL
Methanol	80%	200 mL
**Total**	**N/A**	**1 L**

N/A = not applicable.

**CRITICAL:** Carcinogenic, mutagenic or toxic properties.

***Note:*** Weigh crystal violet under a fume hood without aspiration, then transfer into a glass bottle. Turn on the fume hood aspiration. Carefully add Milli-Q water before adding methanol. Store at RT, away from light for up to 1 year.

## Step-by-step method details

### Slicing procedure

**Timing: About 10 min for steps 1–3; 30 min for steps 4 and 5 (depending on the size of the resection); and finally 30 min for the last steps 6–10**

In this step, we describe how to start the slicing procedure (Figure 1A) to ensure reproducible generation of uniform huOLCs from lung resection, which is essential for preserving tissue viability and structural integrity for at least five days.

1. Collect the lung tissue samples immediately following surgical resection (performed at the CHRU of Tours or the HCL of Lyon) and transfer into Hibernate®-A containing primocin 0.1 mg/ml (or Penicillin/streptomycin) or in Celsior solution.
2. Transfer the lung resection into a large petri dish containing slicing medium.

***Note:*** Gently rinse the resection to remove blood in the slicing medium.

3. Rinse the tissue by simply placing the resection in the slicing medium without using a pipet or any additional tool.
4. Transfer the lung resection to another petri dish containing enough slicing medium to cover the resection.
5. Carefully remove the pleura using a scalpel to facilitate slicing. Trim the lung resection if necessary.
6. Use a sterile 6-mm biopsy punch fitted with a pipette bulb to generate cylindrical tissue cores from the lung resection.

**CRITICAL:** This step should be performed by two people; one person stabilizes the lung resection using tweezers while the other one extracts the sample with the biopsy punch.

7. Place each “core tissue sample” in a 35-mm petri dish filled with 5 ml slicing medium. Let the core tissue sample gently elongate for up to 5min to ensure a proper form for slicing.
8. Once all the core tissue samples are prepared, transfer one tissue core onto 4 autoclaved Whatman papers, and humidify the core tissue with a drop of slicing medium.
9. Place the Whatman papers onto the slicing platform of the McIlwain tissue chopper.

**CRITICAL:** The tissue chopper blade is extremely sharp. Use caution when placing and adjusting samples.

10. Perform the slicing (500 μm). See Troubleshooting problems 1 and 2.
11. Transfer the Whatman paper with the slices to a new 35-mm petri dish with 5 ml of fresh slicing medium.
12. Carefully remove the human lung organotypic slices using curved sterile tweezers and separate the lung slices.
13. Remove all the Whatman paper from the petri dish.

***Note:*** The number of successful huOLCs per tissue sample will depend on the size of the resection (particularly its thickness) and the anatomical area (more or fewer pleura, dark areas). We can usually obtain between 5 and 10 per biopsy punch of 1 cm thick tissue. More details are presented in Figure 1B.

### Transfer onto inserts and culture procedure

**Timing: About 10 min for steps 14–16 and 20 min for steps 16–20 (variable, depending on the number of slices)**

These steps describe how to correctly place tissue cultures on the insert to enable the efficient exchange of nutrients and gas at the air-liquid interface, thereby reducing hypoxia and maintaining tissue viability. The plating procedure is essential to ensure uniform exposure to viral inoculum and/or treatment to preserve reproducible infection/treatment efficiency.

14. Inside a safety cabinet carefully remove the medium under the insert using a P1000 Pipetman with filter tips
15. Add 1 mL of fresh medium under the insert before depositing the human organotypic lung slices.
16. Replace the 6 well plate into the incubator under a humidified atmosphere of 5% CO_2_ and 37°C.

**CRITICAL:** This must be completed prior to the beginning of the slicing procedure to avoid drying of the insert membrane and to allow immediate plating of generated slices.

17. Select slices for the culture under the stereomicroscope. Note: slices must be uniform in thickness and round in shape without signs of folding or tearing. Depending on the addressed question, avoid using cultures presenting signs of fibrosis or tar deposits.
18. Carefully place between 2 to 6 slices per insert membrane using the 5 mL truncated pipette mounted on a pipette bulb.

***Note:*** huOLCs should be placed on the insert as presented in the schematic below without touching each other or the edges of the membrane, as contact with the edges can alter infection patterns and affect the viability of huOLCs (Figure 3).

19. Gently remove the excess of slicing medium on the membrane by using a P200 Pipetman avoiding displacement of slices.
20. Incubate the slices at 37°C, under 5% CO_2_ in a humidified atmosphere. See Troubleshooting problems 3 and 4.

**Figure 3:**
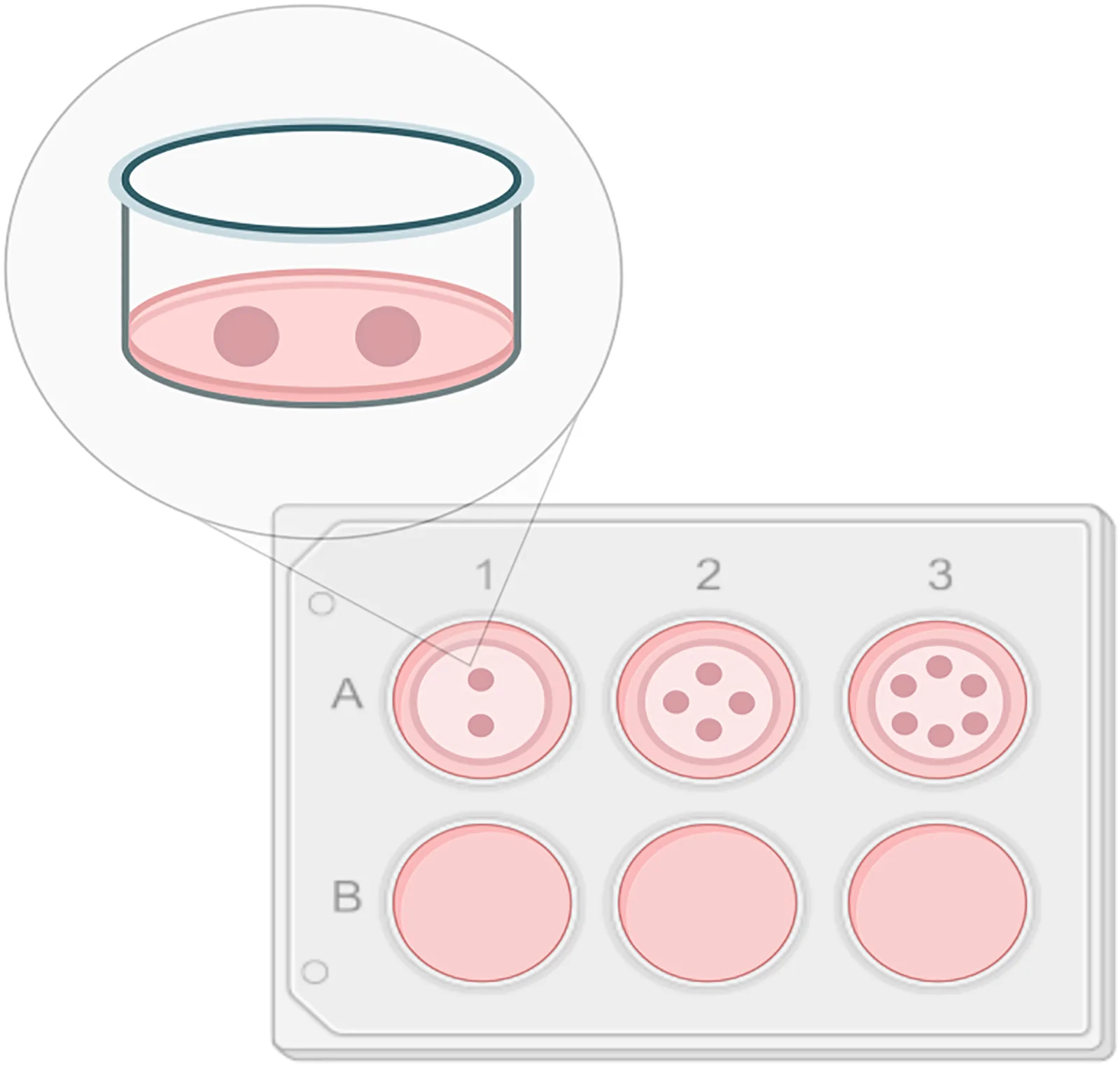
Schematic of the organization of the slices onto the insert in the 6well plate

### Viability procedure

**Timing: MTT (3-(4,5-dimethylthiazol-2-yl)-2,5-diphenyltetrazolium bromide) Cell Viability Assay (steps 21a–e), about 5 h (including the incubation time)**

LDH-Glo Cytotoxicity assay a-d about 30 min including LDH storage buffer preparation and 1 h 10 min for steps e-k.

Cells viability live/dead assay a-r about 1h30min until cytometry monitoring.

Cells viability in huOLCs by fluorescence microscopy a-e about 2 h until microscopy observation.

In this step, we assess tissue viability using complementary methods of *(3-(4,5-dimethylthiazol-2-yl)-2,5-diphenyltetrazolium bromide)* MTT and Lactate dehydrogenase (LDH) assays, to monitor cellular integrity and metabolic activity. MTT is used as a proxy for tissue viability using quantitative viability assays while LDH quantifies tissue damage by measuring the release of cytoplasmic enzymes into the culture medium. We have completed this study with a flow cytometry method using LIVE/DEAD staining, and a microscopic method using propidium iodide to visualize cell death directly on the tissue counterstained with Hoechst. These last two methods make it possible to evaluate the percentage of cell death among total cells per observed section.

21. MTT Cell Viability Assay (see Figure 4A)
  a. Assess the viability of the human lung organotypic slices at 0, 24, 48, and 72 h timepoints.
  b. The MTT used is a Sigma Aldrich powder (1 g), reconstituted in sterile 1X DPBS under BSC to achieve the final working concentration of 5 mg/ml.
  c. Transfer one huOLC per well into a 96-well plate and add 70 μL of MTT solution incubate for 4 h at 37°C. Alternatively, prepare a separate plate fully dedicated to viability assay. In this case either immerse slices into MTT solution or directly add 70 μL of MTT solution on the top of each slice to fully cover the huOLC to ensure total exposure of the tissue.
    **CRITICAL:** MTT is toxic for the skin and eyes and must be handled in a chemical fume hood and BSL-2/4 conditions are required when working with viruses. Wear gloves and protective eyewear.
    ***Note:*** MTT can be prepared in advance, stored at 4°C protected from light for up to 3 weeks.
  d. Transfer each slice into a well of a 48-well plate and cover with 200 μL of DMSO, and triturate to solubilize formazan crystals (violet solution).
  e. After 10 min incubation at 37°C, transfer 75 μL of DMSO containing formazan, without tissue pieces in a transparent 96-well plate to measure absorbance at 540 nm with a microplate reader (e.g., Tristar 5, Berthold).

**Figure 4:**
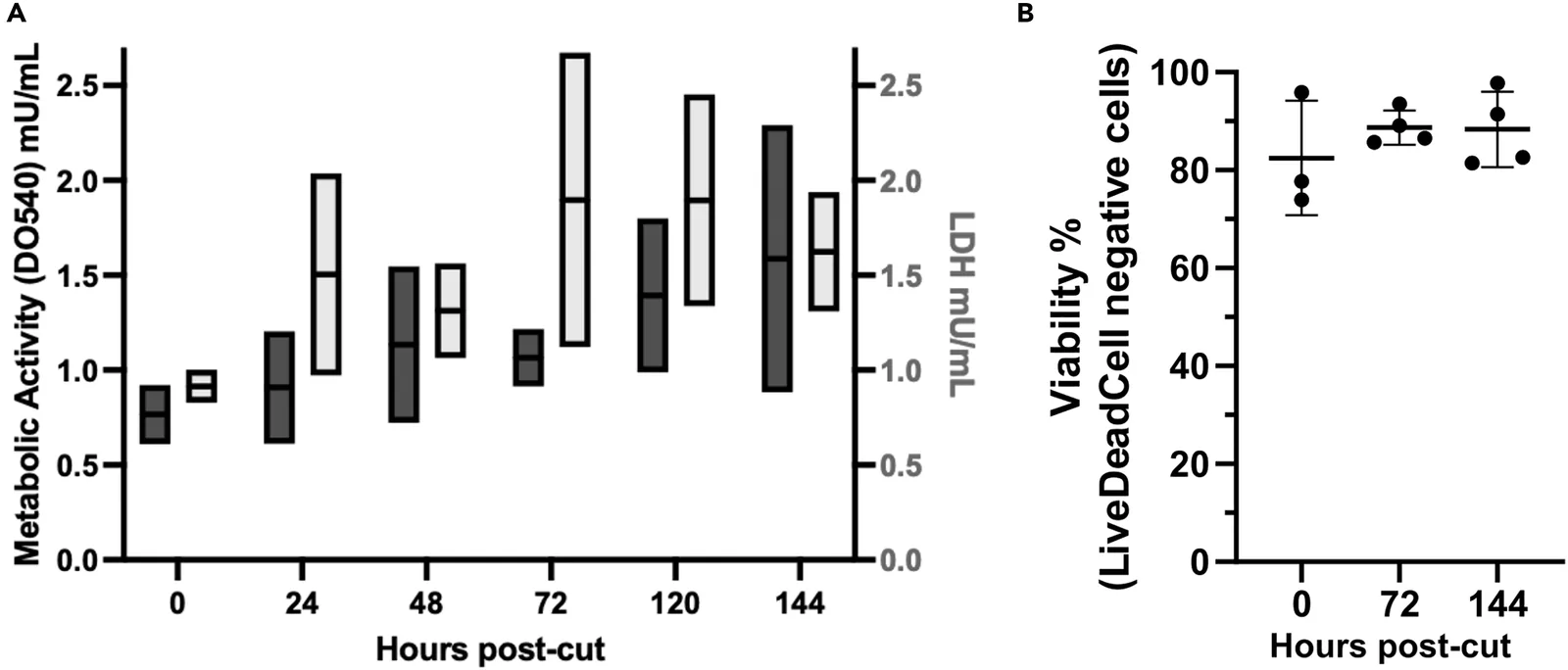
Viability of huOLCs (A) Metabolic activity of huOLCs was measured by MTT assay at 0, 24, 48, 72, 120 and 144 h post-culture (N=2 separate donors) (in black) and LDH release in subnatants was quantified 0, 24, 48, 72, 120 and 144 h of culture (N=2 separate donors) (in gray). (B) Viability of huOLCs was analyzed by LIVE/DEAD Cell labelling at 0, 72, and 144 h of culture (Two separate donors: one sampled at 0 and 72 h, the other at 72 and 144 h). Results are presented as means +/- standard deviation.
22. LDH-Glo Cytotoxicity assay (Figure 4A).
  To assess Lactate dehydrogenase (LDH) release in subnatants at 0, 24, and 48 h of culture.
  a. Prepare LDH storage buffer according to manufacturer instructions (see reagents setup section).
  b. Reconstitute LDH with 275 μL of Storage Buffer and mix to make a 1,000 U/mL standard. The LDH standard is aliquoted at −20°C.
  c. Collect 1.5 μL of homogenized subnatant into 148.5 μL of LDH storage buffer to preserve LDH.
  d. Store aliquots at −20°C until day of the assay.
    The day of LDH quantification.
  e. Thaw samples, LDH detection enzyme mix, LDH storage buffer and LDH standard at ∼25°C.
  f. Prepare LDH standard dilutions as follows:
    i. Dilute 10 μL of LDH standard in 3.115 mL LDH storage Buffer to reach 3.2 U/mL LDH final concentration.
    ii. Mix 10 μL of 3.2 U/mL LDH standard with 990 μL of LDH storage buffer to reach final concentration of 32 mU/mL.
    iii. Serially dilute 1:2 the 32 mU/mL LDH standard solution by mixing 200 μL of previous concentration with 200 μL of LDH storage buffer until reaching LDH final concentration of 0.5 mU/mL.
  g. Prepare LDH detection reagent by mixing LDH Enzyme Buffer Mix and Reductase: for one reaction, mix 50 μL of LDH assay with 0.25 μL of reductase.
  h. Add 50 μL of sample or standard in a white 96-well plate.
  i. Add 50 μL of detection reagent buffer per well.
  j. Incubate the plate at ∼25°Cfor 30 min.
  k. Measure Luminescence using Tristar 5 reader (e.g., Berthold).
23. Cells viability analysis (Figure 4B)
  a. For each donor pool 2 to 3 slices to ensure having enough cells in total (at least 120,000 cells).
    ***Note:*** For count divide the final result by the number of pooled slices.
  b. Incubate each pool for 30 min at 37°C in 500 μL of digestion buffer.
  c. Dissociate cells with approximately 20 aspiration-displacement cycles with a 20G needle.
  d. Filtrate with 40 μm sieve. Wash the sieve three times with DPBS 1X/5% FBS to limit cell loss.
  e. Incubate for 4 min at ∼25°C with 1 mL of Red Blood Cell Lysis Buffer.
  f. Add 9 mL of DPBS 1×/5% FBS.
  g. Centrifuge 5 min at 500 × *g*.
  h. Resuspend in DPBS 1×/5% FBS, 2 mM EDTA.
  i. Centrifuge 96-well plate 5 min at 400 × *g* and 4°C.
  j. Meanwhile, dilute the LIVE/DEAD cell reagent mixture 1000 times.
  k. Remove the supernatant by inversion.
  l. Resuspend the cells with 50 μL of LIVE/DEAD cell reagent mixture.
  m. Incubate 25 min at 4°C.
  n. Add 150 μL of DPBS 1×/5% FBS, 2 mM EDTA.
  o. Centrifuge 96-well plate 5 min at 400 × *g* and 4°C.
  p. Remove the supernatant by inversion.
  q. Resuspend the cells with 200 μL of DPBS 1×/5% FBS, 2 mM EDTA.
  r. Performed the flow cytometry using CytoFLEX cytometer (Beckman Coulter) (without acquisition time).
24. Cells viability in huOLCs by fluorescence microscopy (Figure 5)
  a. The day of interest collect for each donor at least one slice.
  b. Slices were immersed in propidium iodide 10 μM solution for 1 h.
  c. After 3 washes in 1× DPBS, slices were fixed for 1 h with 4% methanol-free PFA.
  d. After 3 washes in 1× DPBS, mount the slices between slide and coverslip using Fluoromount mounting medium containing 1:1 000 of Hoechst solution.
  e. Observe fluorescence.

**Figure 5:**
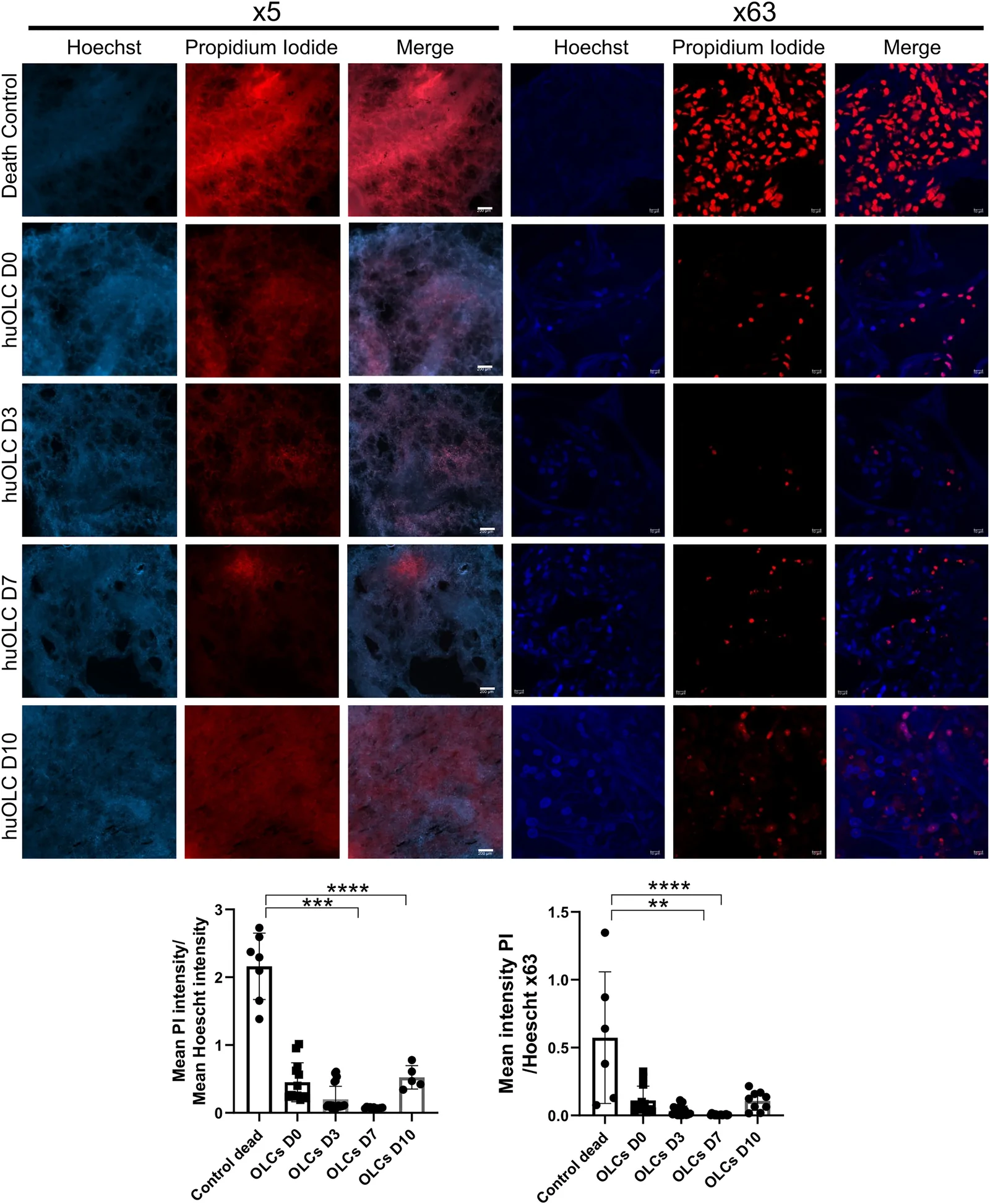
Assessment of cell viability in huOLCs by fluorescence microscopy Lung slices were stained with propidium iodide (PI) to label dead cells and Hoechst to visualize nuclei. Representative fluorescence images at day 0, 3 and 10 post-cut acquired at ×5 and ×63 magnifications are shown. Quantification of cell death was performed by measuring PI fluorescence intensity normalized to the Hoechst signal. Viable huOLCs were compared to a positive control condition in which slices were subjected to heat treatment (70 °C for 5 min) followed by freezing at −20 °C for 5 min to induce cell death (Control dead). These huOLCs were derived from two experiments conducted using two independent patients. For each patient, two huOLCs were used for each condition. Image analysis was performed using Fiji and Zeiss software. Statistical analyses were performed using the Kruskal-Wallis with Dunn’s multiple comparisons test. Statistical significance: ∗p < 0.05, ∗∗p < 0.01, ∗∗∗p < 0.001, ∗∗∗∗p < 0.0001.Results are presented as means +/- standard deviation.

***Note:*** Once mounted, immune-stained slices can be stored at 4°C at least several weeks.

### Cytoflex count

**Timing: 40 min for digestion (step 23) and about 20 min for steps 25–27**

This step consists of dissociating the huOLCs to recover their constituent cells, which are subsequently quantified by flow cytometry (Figure 6).

**Figure 6:**
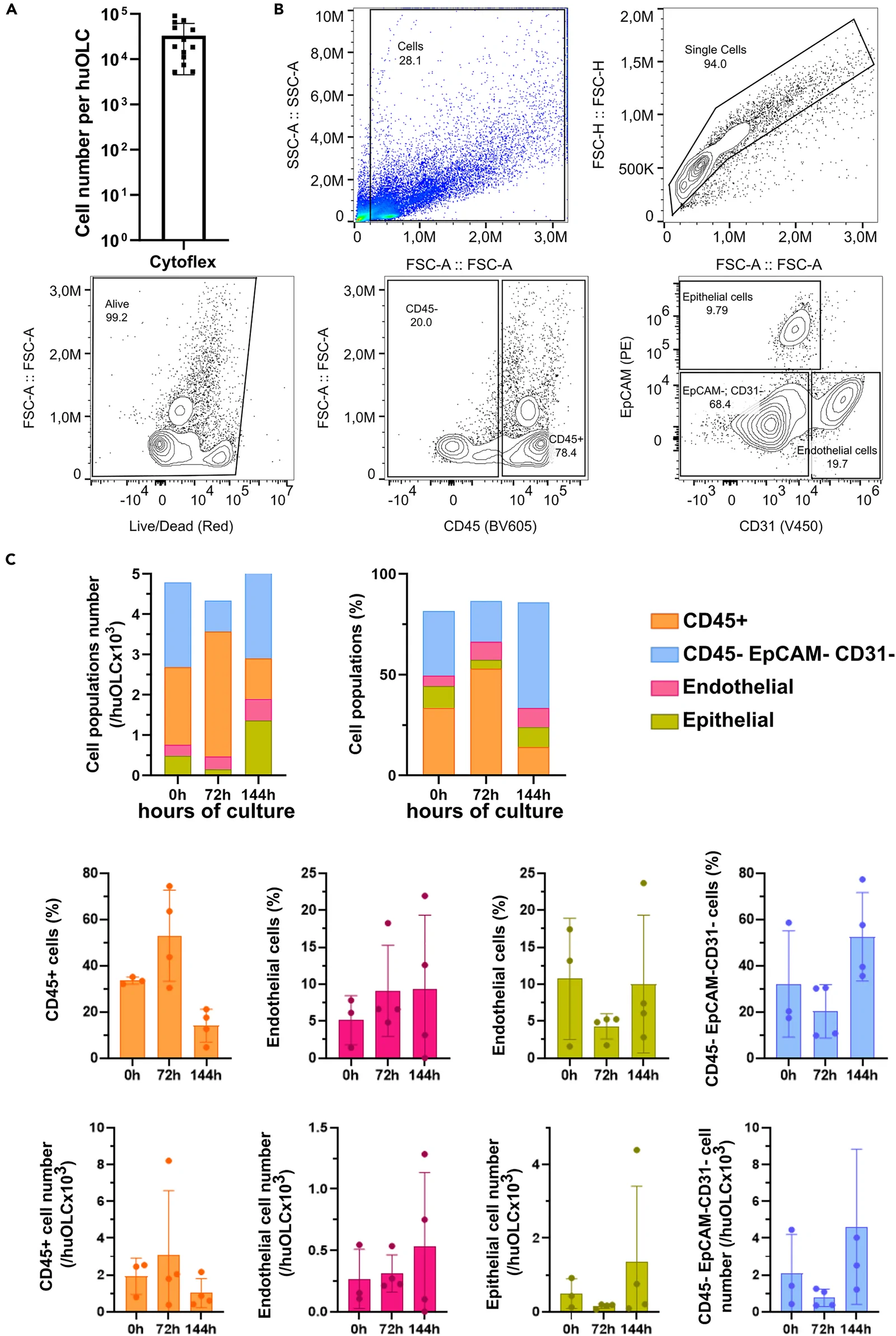
Cells analysis (A) huOLCs were digested for cell dissociation, and cell counting was performed using CytoFLEX at 0 h post-cut.Results are presented as mean +/- standard deviation. (B) Gating strategy used for the characterization of cell populations in huOLCs. (C) The proportions and numbers of cellular populations present in huOLCs at 0 h, 72 h, and 144 h post-cut were evaluated using CytoFLEX. A pool of at least two huOLCs was used for each condition, derived from four independent experiments using tissues from distinct patients.Results are presented as means +/- standard deviation.

Starting from step h. (or step q. if LIVE/DEAD is preferred in all staining) described above in “Cells viability analysis”, this step consists of quantifying by flow cytometry the cells recovered from dissociated huOLCs (Figure 4B). Resuspend the cells with 200 μL of DPBS1×/5% FBS, 2 mM EDTA.

25. Perform the flow cytometry using CytoFLEX cytometer (Beckman Coulter) (without acquisition time).
26. Count cells using flow cytometry-based Precision Count Beads (BioLegend) according to the manufacturer’s instructions.
27. The data were analyzed with FlowJo v.10 (BD Biosciences).

***Note:*** Only include huOLCs that are viable in the assay.

### Cell phenotype analysis

**Timing: About 8 min of cell preparation (steps 28–30); 25 min of incubation (step 31); 8 min of wash (steps 32–34); and steps 35 and 36, depending on the number of wells(count at least 5min per well)**

Resuspended cells are stained with fluorophore-conjugated antibodies targeting population-specific markers. Single-cell fluorescence is then analyzed by flow cytometry, allowing identification and quantification of the distinct cellular populations present within the huOLCs.

28. After step i) described above in “Cells viability analysis”, cells were stained as described below for flow cytometry.

***Note:*** Prepare the antibody mixture (the antibodies are listed in Table 2).

29. Remove the supernatant of centrifuged plate by inversion.
30. Resuspend the cells with 50 μL of antibody mixture.
31. Incubate 25 min at 4°C.
32. Add 150 μL of DPBS 1×/5% FBS, 2 mM EDTA.
33. Centrifuge 96-well plate 5 min at 400 × *g* and 4°C.
34. Remove the supernatant by inversion.
35. Resuspend the cells with 200 μL of DPBS 1x/5% FBS, 2 mM EDTA.
36. Perform the flow cytometry with CytoFLEX instrument (Beckman Coulter) (without acquisition time).

***Note:*** The data were analyzed with FlowJo v.10 (BD Biosciences). The number of cells was determined using flow cytometry–based Precision Count Beads (BioLegend) according to the manufacturer’s instructions.

**Table 2:** Antibodies and LIVE/DEAD Cell reagent used for cell phenotyping

Antibody	Fluorochrome	Dilution used	Cell population
EpCAM	PE	1/200e	Epithelial cells
CD31	VB (BV421)	1/200e	Vascular endothelial cells
CD45	BV605	1/400e	Innate lymphoid cells
LIVE/DEAD Cell	ECD	1/1000e	Cell death

### Infection procedure

**Timing: About 30 min for steps 37–39 (depending on the number of slices)**

This section describes the viral infection of huOLCs by Influenza A virus (IAV) and recombinant Nipah virus (Malaysia strain), expressing the gene encoding the EGFP (rNiV-EGFP), under controlled conditions to ensure efficient viral entry and preserving tissue viability (Figure 7). Precise parameters are essential for reproducible downstream analyses.

***Note:*** All procedures described in the following protocols are performed in biological safety cabinets under sterile working conditions, while wearing personal protective equipment in accordance with BSL-2 and BSL-4 regulations for IAV and NiV, respectively. Refer to your local regulations and permits to determine what biosafety level is appropriate for your strains.

37. Dilute the viral stock in sterile MEM medium for A/Scotland/20/74 (H3N2) or in sterile Opti-MEM medium for rNiV-EGFP to infect the huOLCs.

***Note:*** For both viruses, FBS free medium is used.

**CRITICAL:** Follow the good laboratory practices and personal protective equipment.

38. Infect the huOLCs with 2,000 to 20,000 plaque forming units (pfu) of A/Scotland/20/74 (H3N2) (BSL-2) or 5,000 or 20,000 pfu of rNiV-EGFP (BSL-4). Using low-binding 10 μL pipette tips, dispense a drop of 4 μL of the virus directly on the slice until the collection time point without removing or washing the viral inoculum.

***Note:*** For the mock control slices, apply the same volume of MEM or Opti-MEM as for the infection.

**CRITICAL:** Ensure that the virus drop is completely and homogeneously distributed on the slice without damaging the tissue.

39. Incubate the infected slices at 37°C under 5% CO_2_ in a humidified atmosphere.
40. For rNiV-EGFP, the fluorescence resulting from viral replication can be followed using a Leica DMIRB microscope, a fluorescence microscope (BSL-4) and Nikon Eclipse Ts2R optical microscope in BSL-2.

**Figure 7:**
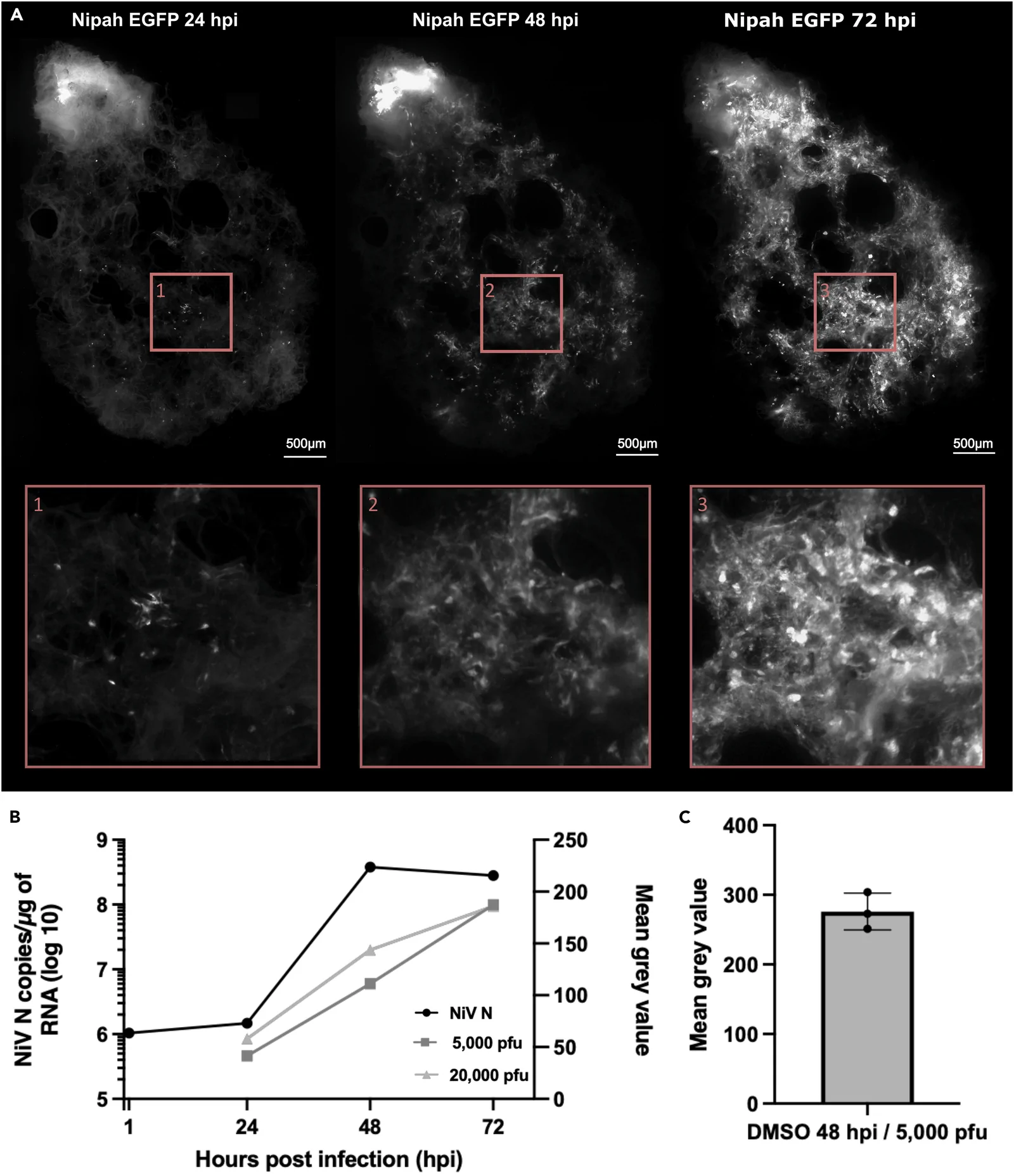
Susceptibility of huOLCs to NiV EGFP (A) Representative fluorescence images of huOLC infected with 5,000 pfu of recombinant NiV EGFP. Images were taken at 24, 48 and 72 hpi using a Leica DMIRB microscope at 5x magnification. Exposure times were 2,000 ms for pictures at 24 hpi and 200 ms for pictures at 48/72 hpi. Scale bar = 500 μm. (B) Infection kinetics represented with the NiV replication kinetics in huOLCs quantified at 1, 24, 48 and 72 hpi (5,000 PFU of rNiV-EGFP). huOLCs were lysed and total RNA extracted, and viral replication was quantified by RT-qPCR targeting the NiV nucleoprotein (N) gene. In parallel, EGFP expression was determined by fluorescence microscopy and results show the mean grey value of huOLCs infected with 5,000 PFU or 20,000 PFU at 24/48/72 hpi. Threshold setting: 90–65565, mean grey value obtained on a specific ROI. (C) Mean grey value of huOLCs infected with 5,000 PFU NiV EGFP at 48 hpi. Threshold setting: 90–65565, mean grey value obtained on a specific ROI. Data represent mean N=3 +/−SD.

### Extraction and RT-qPCR procedure

**Timing: For huOLCs infected by NiV (steps 41 and 42): 30 min (including 10 min of TissueLyser); step 43: 2 h (1 h for the extraction + 30 min rDNAse + 30 min for RNA quantification); step 44: 30 min for the preparation of the RT and 26 min for the RT run; step 45: 30 min for the preparation of the qPCR and 2 h for the qPCR run**

For huOLCs infected by IAV: 41–42: 20 min (including tissue lysis using Precellys apparatus); 43: 2 h (1 h for the extraction + 30 min rDNAse + 30 min RNA quantification); 44: 30 min for the preparation of the RT and 1 h 15 min for the RT run; 45: 30 min for the preparation of the qPCR and 1 h 15 for the qPCR run.

These steps detail RNA extraction from infected huOLCs and subsequent RT-qPCR analysis to quantify viral and host gene expression (Figure 8). Standardized extraction and RT-qPCR conditions are critical for accurate and reproducible quantification.

**Figure 8:**
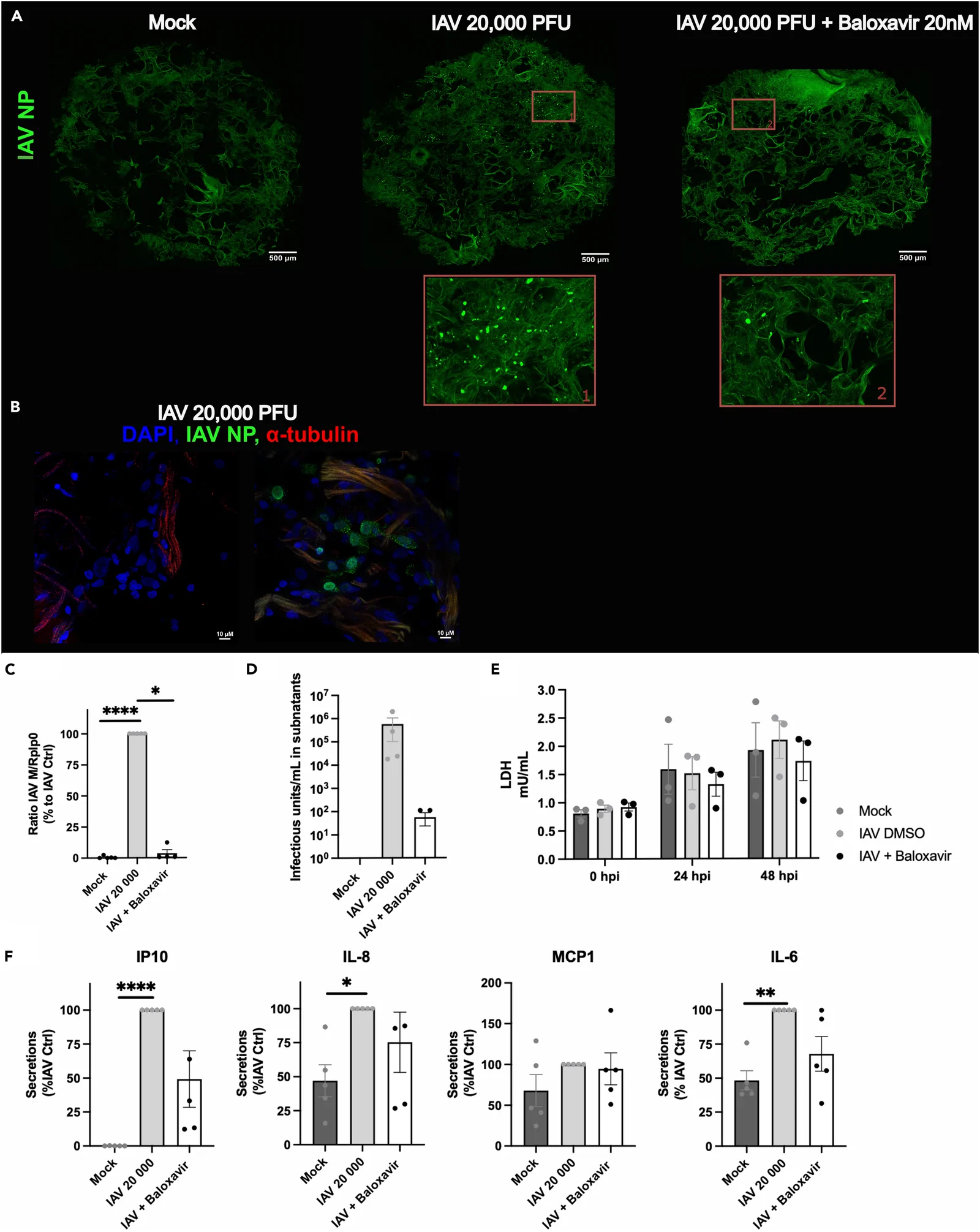
Susceptibility of huOLCs to IAV H3N2/Scotland strain (A) Representative immunofluorescence images showing IAV nucleoprotein expression at 48 hpi, in huOLCs that were either not infected, infected with 20,000 pfu of A/Scotland/20/74 (H3N2) virus, or infected and treated with 20 nM Baloxavir. Images were taken using a spinning Disk confocal microscope CQ1 Yokogawa CQ1 confocal microscope, at x10 magnification. Exposure times were 0.5 s. Scale bar = 500 nm. (B) Confocal microscopy analysis of IAV-infected huOLCs at 48 hpi following infection with 20,000 pfu of Influenza A/Scotland/20/74 (H3N2) virus. Viral nucleoprotein (green), nuclei (blue) and α-tubulin (red) are shown. huOLCs were stained for 12 hours at 4°C with anti-Tubulin (1/500) and anti-NP-FITC (1/50) antibodies. The anti-mouse-Alexa Fluor 647 secondary antibody was applied for 2 h at 25°C for α-tubulin staining. Images were acquired using a Leica SP8 confocal microscope at x63 magnification, exposure time 1 to 2 min and 3D reconstructions were generated with Leica LasX Life Sciences Software. Scale bar = 10 μm. (C) IAV replication in huOLCs at 48 hpi was quantified by RT-qPCR targeting the viral Matrix (M) segment and normalized to the housekeeping gene Rplp0 gene following tissue lysis and RNA extraction. (D) Quantification of infectious viral particles in huOLCs at 48 hpi following infection with 20,000 pfu of A/Scotland (H3N2) strain, in the presence or absence of 100 nM of Baloxavir. Viral production was determined by TCID50 titration on MDCK cells. (E) Measurement of huOLCs viability by quantification of lactate dehydrogenase (LDH) secretion into the culture medium, as a proxy of cell death. Supernatants were collected at 0, 24, and 48 hpi. (F) Quantification of cytokine and chemokine secretion in the basolateral compartment at 48 hpi. HuOLCs were infected with 20,000 pfu of IAV A/Scotland/20/74 (H3N2) strain and treated or not with 100 nM Baloxavir. Cytokines and chemokines were quantified using bead-based immunoassays (CBA). For All graphs, results are presented as means +/- standard deviation.

Two slices are collected per condition and lysed together to ensure sufficient material for RT-qPCR.

41. Collect the huOLCs at 0, 24, 48, and 72 hours post infection (hpi).
42. Transfer 2 slices per condition into a 2 mL Eppendorf tube containing:
  a. 1 mL of Trizol and 2 stainless beads (5 mm diameter) for rNiV-EGFP.
  b. 800 μL of Trizol and 4 stainless beads in Precellys Bertin 2 mL tubes for IAV infection.
43. huOLC lysis protocol:
  a. For rNiV-EGFP: Use a TissueLyserII (Qiagen) at maximum speed for 10 min to fully dissociate the tissue.
  b. For IAV: Use a Precellys homogenizer with the following protocol performed 3 times: 3 x (15 s at 5196x g– 15 s break).
44. Extract total RNA using the Direct-zol RNA Miniprep Kit (Zymo Research), following the manufacturer’s procedure.

**CRITICAL:** Store purified RNA at −80°C to prevent degradation.

45. Synthetize cDNA with 50 ng of extracted RNA using:
  a. For rNiV-EGFP: the iScript cDNA Synthesis kit (Bio-Rad) according to the manufacturer’s protocol.
  b. For IAV: the High-Capacity RNA-to-cDNA kit (Thermo Fisher) according to the manufacturer’s protocol.
46. cDNA are diluted 1/10.

**CRITICAL:** Store the cDNA at −20°C.

47. Perform the qPCR with the Platinum SYBR Green qPCR SuperMix-UDG (Invitrogen) using a StepOne Plus Real-Time PCR System (Applied Biosystems) according to the manufacturer’s protocol.

***Note:*** Use the qPCR primers designed by Beacon Designer (version 8) software or chosen after validation such that their efficacy is close to 100% according to the MIQE checklist.^29^ GAPDH (Glyceraldehyde 3-phosphate dehydrogenase) and human Rplp0 (Ribosomal Protein Lateral Stalk Subunit P0) are used as housekeeping genes. Run all samples in duplicates and analyze results using StepOne version 2.3 (Applied Biosystems). Normalize all results to the standard deviation (SD) for GAPDH mRNA using GAPDH primers^27^ or Rplp0^10^^,^^30^ mRNA and do all calculations using the 2^−ΔΔCT^ model.^31^ Perform all the statistical analyses using GraphPad Prism v10.

### Fixation and immunostaining procedures for viral detection

**Timing: 10 min for the fixation (step 48); about 1 h 30 min for steps 49–56; 10 min + 12 h for step 57; 40 min + 12 h for steps 58–60; and 1 h for the last step**

In this step, we describe fixation and immunostaining procedures used to visualize viral antigens. By staining huOLCs after fixation it is possible to determine whether the infection is present or absent.

48. Fix slices infected with rNiV-EGFP with 4% methanol-free paraformaldehyde (PFA) for 14 days.
  a. Fix slices infected by A/Scotland/20/74 (H3N2) for 2 h with 4% methanol-free PFA.

**CRITICAL:** Carcinogenic, mutagenic or toxic properties. Handle in a fume hood with personal protective equipment.

49. Wash slices 3 times with 1X DPBS, 5 min per wash to remove excess PFA.
50. Gently transfer the slices into a 48-well plate using the 5 mL truncated pipette mounted on a pipette bulb.
51. Saturation of aldehyde groups: add 200 μL/well of 1X DPBS + 0.1% glycine, incubate for 10 min at RT.

**CRITICAL:** Always check that no bacteria or fungi are present in solutions prior to use.

52. Wash the slices with 1X DPBS.
53. Permeabilize and block non-specific binding sites with 200 μL/well (1X DPBS + 0.5% Triton X-100 + 1% BSA for 2 h at ∼25°C or for 12 hours at 4°C).
54. Wash slices in 1X DPBS.

**Pause point:** Slices can be stored at 4°C.

55. Add the primary antibody diluted as recommended in blocking solution and incubate for 12 hours at 4°C with a gentle shaking.

***Note:*** For IAV-infected huOLCs, viral nucleoproteins were stained for 12 hours at 4°C, using a rabbit anti-Influenza nucleoprotein primary antibody diluted 1:400 in blocking solution.

56. Remove the primary antibody solution and wash 3 times in 1X DPBS for 5 min.
57. Dilute the secondary antibody in blocking solution and incubate slices for 12 hours at 4°C.

***Note:*** For IAV-infected huOLCs, the slices were incubated for 2 h at ∼25°C, with goat anti-rabbit Alexa 488 conjugated secondary antibody diluted 1:1000 in blocking solution.

**Pause point:** The secondary antibody can be left 2 h at ∼25°C.

58. Wash 3 times: 2 fast washes with 1× DPBS (5 min each) and one long wash (15 min).
59. Incubate huOLCs for nuclei staining for 45 min at ∼25°Cwith Hoechst solution diluted 1:1 000 in blocking solution.
60. Mount the slices between slide and coverslip using Fluoromount mounting medium.
61. Observe fluorescence (see Troubleshooting Problem 5).

***Note:*** Once mounted, immunostained slices can be stored at 4°C at least several weeks.

### Titration of IAV infectious particles by TCID_50_ assay

**Timing: Step 62: 5 min; step 63: 35 min (time can vary according to the number of plates); steps 64–66: 2 days and approximately 1 h 30 min to 2 h depending on the number of plates; step 67: 2 days; steps 68–69: 30 min; and step 70: 10 min**

This step aims to measure the concentration of infectious viral particles secreted in the subnatants of huOLCs using TCID_50_ titration assay in which serial dilution of huOLC subnatants are used to infect MDCK cells. The resulting TCID_50_ values determine infectious viral titers and confirm that the huOLC model is permissive to IAV infection and supports effective viral replication in various conditions (time, treatment etc.).

62. Subnatants dedicated to titration are aliquoted and stored at −80°C until the day of titration (see materials and equipment section).
63. MDCK cells are seeded at 11,000 cells/well in 96-well plates, in MDCK complete culture medium.
64. Two days after MDCK seeding, the medium is removed and MDCK cells are washed twice with 1X DPBS.
65. Subnatants are serially diluted (10-fold) in MEM infection medium (see materials and equipment section).
66. 1X DPBS is removed, and cells are inoculated with each dilution (total of 8 replicates for each subnatant dilution).
67. MDCK cells are incubated for 2 days at 37°C, 5% CO_2_.
68. Medium is then removed, and cells are fixed and stained with 0.3% crystal violet + 20% methanol solution for 20 min (see materials and equipment section).
69. Crystal violet solution is removed, and cells are rinsed three times with 1X DPBS.

**CRITICAL:** Carcinogenic, mutagenic or toxic properties. Handle in a fume hood with personal protective equipment.

70. Viral titer is determined using the Reed and Muench method.^32^

### Cytokine and chemokine quantification

**Timing: Step 71: 5 min; steps 72 and 73: 5 min; step 74: 30–35 min ± depending on the nature, number, and dilution of samples; step 75: 1 h 40 min; and step 76: 3 h ± depending on the number of samples for the acquisition on flow cytometer**

This section describes a protocol to quantify cytokines secretions in huOLC subnatants over time and/or in various conditions (treatment) using BD Cytometric beads array (CBA) for Human Soluble Proteins Flex Set assay according to the manufacturer instructions.

71. Aliquot and store huOLC subnatants at −80°C.

The day of the assay:

72. Humidify the 96-well PALL® filter plate with 100 μL of wash buffer.
73. Aspirate wash Buffer gently using a vacuum manifold.

**CRITICAL:** Do not touch the filter with tips. Do not exceed 10′ in Hg of vacuum pressure. Aspirate for 2 to 10 s until wells are drained.

74. Preparing Human Flex Set Standards:
  a. Preparation of the top standard: Mix lyophilized standards from each BD CBA human Soluble Protein Flex Set in a 15 mL falcon tube with 2 mL assay diluent to reach a 5,000 pg/mL dilution.
  b. Incubate top standard solution for 15 min at ∼25°C.
    **CRITICAL:** Do not vortex.
  c. Dilute standard solution by half by mixing 200 μL of standard with 200 μL assay diluent.
  d. Repeat the 2-fold dilution process until reaching 1:256 dilution, e.g., 8 serial dilution tubes + 1 top standard.
    ***Note:*** Additional dilutions can be performed if required.
  e. Prepare a negative control with assay diluent only.
  f. Distribute 40 μL of standard dilution or negative control per well.
  g. Dilute subnatants in Assay diluent to remain within the standard range for each cytokine.
75. Mixing Human Soluble Protein Flex Set Capture beads:
  a. Determine the number of BD CBA human Soluble Protein Flex Sets to be used, as well as the number of samples (including standards) in the experiment.
  b. Vortex each capture beads stock vial for 15 s to resuspend beads.
  c. For each set, and as many as sample, mix 0.8 μL of each cytokine capture bead complete to 40 μL with Capture beads diluent.
    ***Note:*** The volumes have to be adapted to the number of samples (S) and cytokines (Y) to be assessed. Use a formula to calculate the total volume V according to the number of samples (S). V= Y x 0.8 μL cytokine capture beads x S. We recommend to count the real number of sample + 1 to ensure a dead volume.
  d. Distribute 40 μL of the capture beads mix over 40 μL of sample or standard.
  e. Cover the plate with seal.
  f. Shake the plate for 2 min at 500 rpm.
  g. Incubate for 1 h at ∼25°C, in the dark.
  h. Keep an aliquot of the beads for setup.
76. Preparing Human Soluble Protein Flex Set PE Detection Reagents:
  a. For each set mix and as many as sample, mix 0.8 μL of each Human Soluble Protein Flex Set PE detection reagent complete to 40 μL with detection reagent diluent.
    ***Note:*** As above V= Y x 0.8μL cytokine capture beads x (S+1).
  b. Distribute 40 μL of PE detection mix per well.
  c. Cover the plate with new seal.
  d. Shake the plate for 2 min at 500 rpm.
  e. Incubate for 2 h at ∼25°C, in the dark.
  f. Aspirate on vacuum manifold until wells are drained.
  g. Add 150 μL per well of wash buffer.
  h. Aspirate on vacuum manifold until wells are drained.
  i. Add 200 μL per well of wash buffer.
  j. Shake the plate for 5 min at 500 × *g* to resuspend beads.
  k. Proceed to sample acquisition on flow cytometer (e.g., LSR Fortessa II cytometer, BD Biosciences) according to manufacturer’s instruction (see Troubleshooting Problems 6 and 7).

## Expected outcomes

This protocol enables the generation of standardized, viable, structurally preserved huOLCs that retain the essential architectural and cellular features of the native tissue, making them suitable for a wide range of applications including viral infection studies. A major advantage of this method is the complete elimination of agarose embedding, which significantly reduces preparation time without compromising tissue integrity and preserves resident immune cells such as alveolar macrophages.^33^ Furthermore, the use of a tissue chopper ensures a rapid and highly reproducible slicing process, which constitutes a key strength of the technique (Figure 1A**)**.

HuOLCs prepared using this protocol are expected to maintain high viability for several days in culture. Tissue viability was assessed by measuring metabolic activity using MTT (Figure 4A) (or MTS assays, not shown), and by monitoring LDH release in the culture medium every 24 h (Figure 4A). As a complementary approach, cell death was evaluated by LIVE/DEAD staining (Figure 4B**)** (or TUNEL staining, not illustrated). Together, these assays consistently demonstrate preserved metabolic activity and tissue integrity for up to 6 days (144 h) of culture, validating the suitability of huOLCs as a robust *ex vivo* tissue culture model suitable for infection with human viruses and by extension, with other pathogens such as bacteria or fungi. Such analysis can also be performed in microscopy as shown in Figure 5, by directly staining the slices using propidium iodide and counterstain with Hoechst in order to evaluate percentage of cell death over the time of culture. As shown in Figure 3 and in numerous former studies with other slices, mortality peaks the day of slicing and then strongly decreases. Cell death increases again after 10 days, confirming the good viability of the slices for more than a week.

As shown in Figure 6A, the CytoFLEX-adapted protocol allows quantification of cells recovered from the tissue immediately after huOLCs preparation and throughout the culture period. Various cell populations can be stained and followed over the time of culture (Figure 6 b). As shown in Figure 6C, all population of cells were still represented after 6 days of culture.

One of the primary applications of this model is the infection of human lung slices with viruses spanning biosafety levels from BSL-2 to BSL-4, such as the highly pathogenic Nipah virus (Figure 7) or the more commonly encountered IAV H3N2 (Figure 8). For example, recombinant Nipah virus expressing EGFP (rNiV-EGFP) (Figure 7A), can be used to study the early steps of infection by fluorescence microscopy, leveraging the reporter signal to visualize infected cells and monitor viral spread within the tissue. Time-lapse microscopy further allows longitudinal assessment of tissue morphology alongside the progression of EGFP-associated viral infection (Figure 7A). Viral RNA accumulation in tissue cultures can also be quantified by RT-qPCR. HuOLCs are fully permissive to NiV infection, with viral replication reaching a plateau at approximately 48 hpi in these conditions of infection (Figure 7B). Alternatively, viral growth can be easily assessed by measuring the mean fluorescence intensity from images acquired daily under standardized exposure conditions. This method of analysis confirms the high reproducibility of the infection with rNiV-EGFP between samples, as illustrated at 48 hpi (Figure 7C).

The susceptibility of huOLCs to IAV infection was characterized at 48 hpi using complementary approaches: immunofluorescent detection of viral nucleoprotein, quantification of viral genome replication by RT-qPCR, and titration of infectious particles in culture subnatants by TCID_50_ assays (Figures 8A–8D). As observed in both NiV- and IAV-infected cultures, a degree of intrinsic tissue autofluorescence may be detected during imaging mainly when working with green fluorescence.

Beyond infection studies, huOLCs can be used to evaluate the antiviral activity of diverse therapeutic molecules. For instance, Baloxavir, a well-characterized inhibitor of IAV polymerase, exhibited significant antiviral efficacy at the concentrations of 20 and 100 nM (Figures 8A–8D). Treatment of IAV-infected huOLCs with Baloxavir did not affect tissue viability as indicated by stable LDH secretion over time (Figure 8E). In parallel, inflammatory cytokines (IL-6 and IL-8) and chemokines (IP10 and MCP1) secreted into the basolateral compartment can be quantified over time by ELISA (Figure 8F). This allows not only the characterization of the inflammatory tissue response to infection but also the evaluation of the anti-inflammatory efficacy of the tested treatments. It is important to note that for milder infections, depending on the strain of IAV, substantial donor-to-donor variability in both cytokine and viral quantification can be observed. Baseline cytokine levels vary widely across donors and inevitably influence the magnitude and kinetics of responses measured following infection. Similarly, infection efficiency can differ markedly between donors, likely reflecting individual’s history (e.g., prior infections, vaccination and environmental exposures, *etc.*) compared to the relatively uniform responses observed in syngeneic mouse models. This effect can be particularly pronounced for IAV, for which past infections and vaccinations are suspected to significantly shape immune imprinting and, consequently, the responsiveness of organotypic human lung cultures. Although this inter-individual variability increases experimental complexity and may challenge reproducibility, it also represents a major strength of the model. By capturing the inherent heterogeneity of human infectious history -something largely absents from traditional experimental systems-these human organotypic cultures provide a physiologically relevant platform for evaluating novel therapies and assessing their robustness across diverse human backgrounds.

## Quantification and statistical analysis

All the quantifications and statistical analyses were performed using GraphPad Prism, FlowJo and ImageJ. Details of the number of biological replicas (n) and statistical tests used are indicated in the corresponding figure legends.

Flow cytometry data were analyzed using FlowJo. Cell populations were identified by sequential gating (Figure 6).

Quantification of fluorescence signal for NiV was performed by measuring the mean gray value within manually defined and applied to all images within the same experiment. Background signal was subtracted prior to quantification. When images were acquired using different exposure times, signal intensities were normalized by applying a correction factor corresponding to the exposure time ratio using Process > Math > Divide in ImageJ (∗).

Fluorescence images were processed and analyzed using ImageJ/Fiji. When necessary, tiled images were reconstructed using the stitching plugin.

(∗) DMSO condition - 48 hpi (5,000 pfu): Images were acquired with an exposure time of 300 ms, whereas comparable conditions were acquired at 200 ms. To normalize signal intensities across images, a correction for exposure time was applied. Signal intensities were normalized by dividing the measured intensity values by the exposure time ratio (300 ms/200 ms = 1.5) using Process > Math > Divide in Image J.

Kinetics - 5,000 pfu: For kinetics analyses, images acquired at 24 hpi were obtained with an exposure time of 2,000 ms while at 48 hpi and 72 hpi, images were acquired with an exposure time of 20 ms. Due to these differences, a correction was applied to the 24 hpi images to allow comparison across time points. Signal intensities at 24 hpi were divided by 10 using Process > Math > Divide in Image J.

Kinetics - 20,000 pfu: Similarly, for 20,000 PFU condition, images at 24 hpi were obtained with an exposure time of 2,000 ms whereas images at 48 hpi and 72 hpi, images were acquired at 20 ms. A correction was therefore applied to the 24 hpi images by dividing signal intensities by 10 using Process > Math > Divide in Image J to normalize signal value and enable direct comparison between time points.

## Limitations

A limitation of this procedure is that the lung resections used came from different anatomical areas of the lung which may contain varying proportions of cells, and so influence infection, as well as altered regions, sometimes fibroses or pigmented area such as “black aggregates” linked to exposure to tobacco/pollution/tar. Tissue selection is constrained by the resection area. Each sample comes from a different patient, introducing variability according to the age, environment and pathology of the patient which allows the study to have a diversity of samples. This tissue heterogeneity may influence the permissiveness of huOLCs to infection. It is important to have inclusion and exclusion criteria that will allow a better appreciation of the number of samples to be included for statistical analysis. Multiple parameters could be modified and adapted depending on the question that is addressed: among them testing another less expensive media, different type of membrane for culture, and the control of oxidative stress are parameters which could be implemented. Sterile conditions can also be upgraded using additional sterilization strategies using UV irradiation, ethylene oxide treatment, or autoclaving (depending on material compatibility). While it was not necessary to replace our medium of culture after infection with these specific viruses, this can be performed depending on the following assays.

## Troubleshooting

### Problem 1

Core tissue sample can stick to the chopper blade of the tissue chopper due to the tissue dryness (Step 1).

### Potential solution

Moisten the “core tissue sample” and the Whatman paper.

### Problem 2

If the blade does not cut the Whatman paper, then it will not slice deeply enough the tissue (Step 1).

### Potential solution

It is important to perform a test with the blade before slicing the “core tissue sample”, using only Whatman papers and medium. If the blade does not cut enough, you must change the blade. This phenomenon may also be visible after a few uses of the blade, indicating that you need to change the blade.

### Problem 3

Sometimes, it is possible to see contamination on huOLCs in the form of bacteria and fungi. This contamination can be visible to the naked eye as well as under a microscope. This contamination can be due to the medium but also the air from dissection environment (Step 1).

### Potential solution

To avoid this problem, it might be advisable to perform the dissection under a biosafety cabinet, filter the different media used, clean the room/instruments, in particular the tissue chopper/biopsy punch/bulb, and turn off the ventilation/air conditioning in the dissection room. The biosafety cabinet allows the manipulation of biological samples under sterile and safe condition. It protects the huOLCs from contamination by using HEPA-filtered laminar airflow.

### Problem 4

Sometimes huOLCs are not homogeneous, due to heterogenous “core tissue sample” extraction, which can cause problems during infection and analysis (Step 2).

### Potential solution

It is important to choose “core tissue samples” carefully in order to obtain homogeneous huOLCs. This selection must be done under a stereomicroscope to minimize variability.

### Problem 5

The strong natural autofluorescence of lung tissue can overlap with the antibody conjugated fluorophores emitting between ∼450 and 550 nm.

### Potential solution

Select a fluorophore that is distant to lung natural autofluorescence wavelength.

Use a confocal microscope.

### Problem 6

Cytokines and/or chemokines are not detected into huOLC subnatants. This can be due to sample being too diluted.

### Potential solution

Try various sample dilutions until finding the optimal one.

### Problem 7

Cytokine level in samples is above top standard value. The sample is not diluted enough.

### Potential solution

Try various dilutions of the sample until finding the optimal set up.

***Note:*** Sample dilution can vary depending on the cytokine quantified.

## Resource availability

### Lead contact

Further information and requests for resources and reagents should be directed to and will be fulfilled by the lead contact, Cyrille Mathieu (cyrille.mathieu@inserm.fr).

### Technical contact

Technical questions on executing this protocol should be directed to and will be answered by the technical contacts, Lola Canus (lola.canus@wanadoo.fr), Florentine Jacolin (florentine.jacolin@gmail.com), or Virginie Vasseur (virginie.vasseur@univ-tours.fr).

### Materials availability

This study did not generate new unique reagents.

### Data and code availability

No new data/code was generated in this study. Any additional information required to reanalyze the data reported in this paper is available from the lead or technical contact upon request.

## Acknowledgments

The project was supported by ANR-ASTRID (ViroMetaBlock project (ANR-22-ASTR-002) funded by the Agence Nationale de Recherche and the Agence Innovation Défense (AID) as well as by ISIDORe. This work also received support from the French government through the France 2030 program and the 10.13039/501100003323ANRS | MIE, specific project ANRS-23-PEPR-MIE 0008 (NIPAH-LISA), and the 10.13039/501100003323Agence Nationale de Recherche (Program Fluccinate; ANR-22-CE15-0001-01). L.C. was supported by a PhD fellowship from 10.13039/501100001677INSERM, the 10.13039/501100006021Direction Générale de l’Armement (DGA), and the Agence Innovation Défense (AID). The salary of F.J. was funded by the ANR, specific program Fluccinate (ANR-22-CE15-0001-01). The salary of E.O. was funded by ANRS | MIE, specific project ANRS-23-PEPR-MIE 0008 (NIPAH-LISA).

The ISIDORe research program is co-funded by the European Union and has received funding from the European Union’s Horizon Europe research and innovation program under grant agreement no. 101046133. The views and opinions expressed are those of the authors only and do not necessarily reflect those of the European Union or the European Research Executive Agency (REA). Neither the European Union nor the granting authority can be held responsible for them.

We are grateful to the CHRU of Tours and HCL of Lyon for providing access to human lung resection samples, and to the patients for their informed consents. We also sincerely thank Coralie Mousset and Prof. Gaëlle Fromont-Hankard (CHRU de Tours) for their valuable contributions to the initial experiments using human lung tissue sections. We also acknowledge the P4 Jean Mérieux laboratory for its essential support in biosafety, biosecurity and high-containment experimentation. We thank Dr. Alexandre Lalande and Clémence Jacquemin for their valuable assistance throughout this study. We thank the imagery platform (LYMIC-PLATIM) of SFR Biosciences Gerland-Lyon Sud and especially Elodie Chatre for her help. We also thank the Plate-Forme IBiSA des Microscopies, PPFASB, Université de Tours, and CHRU de Tours. All statistical analyses were performed with GaphPad v.10. Graphical abstract/figures were created using Biorender.com: https://biorender.com/gel9ujl.

## Author contributions

Conceptualization, C.M., M.S.-T., F.A., L.P.-C., and P.-O.V.; methodology, L.C., F.J., V.V., and C.M.; software, L.C., A.B., V.V., A.C., and C.M.; validation, M.S.-T., A.G., A.L., C.D., D.S., L.P.-C., P.-O.V., L.C., F.J., V.V., A.C., F.A., A.E., and C.M.; formal analysis, L.C., F.J., V.V., A.C., C.D., E.O., A.B., A.A.-G., and C.M.; investigation, L.C., F.J., V.V., C.D., A.A.-G., A.C., A.G., M.S.-T., L.P.-C., P.-O.V., and C.M.; resources, M.S.-T., A.G., A.L., A.E., F.A., and V.L.; data curation, L.C., F.J., V.V., C.D., A.A.-G., A.C., A.G., M.S.-T., L.P.-C., P.-O.V., and C.M.; writing – original draft, L.C., F.J., V.V., A.C., A.B., L.P.-C., M.S.-T., A.G., P.-O.V., and C.M.; writing – review and editing, L.C., F.J., V.V., A.C., A.B., L.P.-C., M.S.-T., A.G., A.L., C.D., D.S., V.L., P.-O.V., and C.M.; supervision, C.M., M.S.-T., L.P.-C., and P.-O.V.; project administration, M.S.-T., L.P.-C., A.G., P.-O.V., V.L., and C.M.; funding acquisition, C.M., M.S.-T., L.P.-C., P.-O.V., and V.L. All authors have read, commented, and approved the final manuscript.

## Declaration of interests

The authors declare no competing interests.
